# Differential RELA and GR recruitment to the *BIRC3*/*BIRC2* locus: Molecular insight as to combinatorial regulation by proinflammatory cytokines and glucocorticoid

**DOI:** 10.1016/j.molpha.2025.100073

**Published:** 2025-09-10

**Authors:** Andrew J. Thorne, Alex Gao, Amandah Necker-Brown, Akanksha Bansal, Keerthana Kalyanaraman, Priyanka Chandramohan, Sarah K. Sasse, Anthony N. Gerber, Mahmoud M. Mostafa, Robert Newton

**Affiliations:** 1 Department of Physiology & Pharmacology and Lung Health Research Group, Snyder Institute for Chronic Diseases, Cumming School of Medicine, University of Calgary, Calgary, Alberta, Canada; 2 Department of Medicine, National Jewish Health, Denver, Colorado; 3 Department of Immunology and Genomic Medicine, National Jewish Health, Denver, Colorado; 4 Department of Medicine, University of Colorado, Aurora, Colorado

**Keywords:** Baculoviral inhibitor of apoptosis repeat–containing gene, Nuclear factor-*κ*B, Glucocorticoid receptor, Cytokines, Transcriptional control

## Abstract

In pulmonary epithelial cells, baculoviral inhibitor of apoptosis repeat–containing (BIRC) gene, *BIRC3*, and to a lesser extent, *BIRC2*, mRNAs were upregulated by interleukin (IL)-1*β* and tumor necrosis factor-*α*. Glucocorticoids also induced BIRC3 mRNA and the glucocorticoid receptor (GR) was recruited to GR-binding regions (GBRs) proximal to, and within, *BIRC3* in A549 and BEAS-2B cells. Four such GBRs drove glucocorticoid-inducible, Organon 34517–antagonized, transcription in A549 cells. IL-1*β* and tumor necrosis factor-*α* recruited the nuclear factor (NF)-*κ*B transactivating subunit, v-rel avian reticuloendotheliosis viral oncogene homolog A (RELA), to RELA-binding regions (RBRs) (R4 and R7) upstream of the *BIRC3* and *BIRC2* transcription start sites. These RBRs drove IL-1*β*-inducible transcription that involved NF-*κ*B. Thus, direct regulation of *BIRC3* by GR and *BIRC3*/*BIRC2* by NF-*κ*B is indicated. IL-1*β*-plus-budesonide also recruited RELA to multiple GBRs, whereas GR was recruited to the main IL-1*β*-induced RBR (R4), effects that correlated with positive IL-1*β*/glucocorticoid transcriptional cooperativity or additivity. At the R5 GBR, RELA was not recruited and both GR binding and glucocorticoid-dependent transcription reduced (*infra*-additivity) on cotreatment. Similarly, the R7 RBR barely recruited GR and IL-1*β*-induced transcription showed *infra*-additivity with IL-1*β*-plus-glucocorticoid. R8 recruited GR and RELA primarily with IL-1*β*-plus-glucocorticoid and revealed transcriptional synergy. Thus, GR/RELA-corecruitment yielded positive cooperative and additive transcriptional effects, whereas recruiting one factor alone associated with *infra*-additivity. Furthermore, DNA looping revealed how multiple RBRs/GBRs may integrate to control transcription. Because IL-1*β*- and glucocorticoid-dependent coupregulation of apoptotic/antiapoptotic genes was widespread, the combinatorial recruitment of RELA/GR to regulatory genes, including *BIRC3*, *CFLAR* plus others in the NF-*κ*B pathway, may be critical to cell fate determination in inflammation.

**Significance Statement:**

Identification of functional GR– and RELA–binding regions at the *BIRC3*/*BIRC2* locus explains the upregulation of BIRC3 expression by glucocorticoids and inflammatory cytokines. Cytokine-plus-glucocorticoid cotreatment revealed positive cooperative and additive interactions between GR and RELA, whereas DNA regions binding only one factor showed reduced effects on binding and transcription. These region-specific outcomes, combined with DNA looping between regulatory regions, provides insight as to how factors at multiple DNA regions may integrate their outputs to produce combinatorial regulation of apoptotic/antiapoptotic genes.

## 1 Introduction

The baculoviral inhibitor of apoptosis (IAP) repeat–containing (BIRC) proteins, BIRC3 and BIRC2, are expressed from the *BIRC3* and *BIRC2* genes and are believed to prevent apoptosis,^1^ in part via roles in the regulation of the inflammatory transcription factor, nuclear factor (NF)-*κ*B.^2^ Classically, NF-*κ*B exists as heterodimers of p50, processed from the precursor protein p105 and produced from the *NFKB1* gene, plus v-rel avian reticuloendotheliosis viral oncogene homolog A (RELA) or p65, produced from the *RELA* gene.^3^ Henceforth designated as p50 and RELA, respectively, this NF-*κ*B dimer is activated by proinflammatory cytokines, including interleukin (IL)-1*β* and tumor necrosis factor (TNF) *α*, to induce inflammatory gene expression. NF-*κ*B is also critical for expression of antiapoptotic and protective proteins including cellular fas associated via death domain-like IL-1*β*-converting enzyme-inhibitory protein, product of the *CFLAR* gene, and superoxide dismutase 2, in addition to BIRC3.^4^, ^5^, ^6^, ^7^, ^8^, ^9^ Often referred to as cellular IAPs, BIRC3 and BIRC2 belong to the IAP family that is defined by the presence of up to 3 N-terminal baculovirus IAP repeats.^10^^,^^11^ BIRC3 and BIRC2 also contain carboxy-terminal really interesting new gene E3 domains that possess ubiquitin ligase activity. These variously enable proteasome-mediated degradation of target proteins or generate scaffolds involved in signal transduction.^10^ However, suggested roles for BIRC3 and BIRC2 are multiple and may include signaling from Toll-like receptors,^12^^,^^13^ nucleotide-binding oligomerization domain-containing receptors,^14^^,^^15^ as well as following virus infection.^16^ Although BIRC3 and BIRC2 may also lead to inflammasome activation,^17^ or regulation of interferon regulatory factors,^16^^,^^18^ the most consistent evidence suggests roles in signaling to NF-*κ*B, in particular from TNF receptors.^2^^,^^19^^,^^20^ Furthermore, separate BIRC3 and BIRC2 functions are not generally distinguished and redundancy is often presumed. However, because BIRC3 is highly induced by cytokines such as IL-1*β* or TNF*α*, whereas BIRC2 protein shows constitutive expression,^9^ assumptions of redundancy may prove simplistic.

Differential regulation of BIRC3 and BIRC2 is further highlighted at the expression level. In pulmonary epithelial cell lines and primary cells, BIRC2 mRNA was modestly induced by both IL-1*β* and TNF*α* and this was reduced by glucocorticoid cotreatment.^9^ However, despite these changes there were little or no effect on BIRC2 protein expression. Conversely, BIRC3 mRNA and protein expression were highly induced by IL-1*β* and TNF*α* via a process that involved NF-*κ*B.^9^ BIRC3 mRNA and protein were also modestly increased by glucocorticoids acting via GR and IL-1*β*-, or TNF*α*-induced BIRC3 was variously unchanged or, dependent on the model, enhanced by glucocorticoid cotreatment.^9^ This outcome contrasts with the effects of glucocorticoids on the expression of many other inflammatory genes, where profound repression of IL-1*β*-, TNF*α*-, or other proinflammatory stimulus-induced gene expression is routinely observed.^8^^,^^21^^,^^22^ Thus, *BIRC3* stands out as an NF-*κ*B–regulated inflammatory gene whose expression is not repressed by glucocorticoids.^8^^,^^23^ Indeed, BIRC3 expression is upregulated in asthma and this expression correlated positively with markers of disease severity.^24^ Further, BIRC3 expression is independently induced by glucocorticoids in numerous systems, including human pulmonary epithelial cell lines, primary human airway epithelial cells, airways smooth muscle cells and human lungs following budesonide inhalation.^8^^,^^25^, ^26^, ^27^

The pulmonary epithelium is both active in inflammation and implicated in the pathogenesis of diseases such as asthma.^28^ Furthermore, the epithelium represents a primary target for inhaled glucocorticoid therapies and appears essential for glucocorticoids to control lung inflammation.^29^ In addition glucocorticoids, such as dexamethasone and the inhaled corticosteroid, budesonide, which act on the glucocorticoid receptor (GR; gene symbol NR3C1), and IL-1*β* and/or TNF*α*, which act via NF-*κ*B, all induce BIRC3 mRNA and protein expression in highly differentiated primary bronchial human epithelial cells, A549 pulmonary epithelial cells and bronchial airway epithelial BEAS-2B cells.^9^^,^^25^ Thus, A549 and BEAS-2B cells represent suitable models to interrogate transcriptional control by GR and NF-*κ*B acting at the *BIRC3*/*BIRC2* loci.

## 2 Materials and methods

### 2.1 Drugs, stimuli, and inhibitors

Recombinant human IL-1*β* and TNF*α* (both R&D Systems) were dissolved in PBS containing 0.1% bovine serum albumin (Sigma-Aldrich). Budesonide (gift: AstraZeneca), dexamethasone (Sigma-Aldrich), Organon 34517 (Org34517) (gift: Chiesi Farmaceutici), PS-1145 (N-(6-chloro-9h-pyrido[3,4-b]indol-8-yl)-3-pyridinecarboxamide) (Sigma-Aldrich), ML120B (N-(6-Chloro-7-methoxy-9H-pyrido[3,4-b]indol-8-yl)-2-methyl-3-pyridinecarboxamide dihydrochloride) (SML1174, Sigma-Aldrich), TPCA-1 (2-[(aminocarbonyl)amino]-5-(4-fluorophenyl)-3-thiophenecarboxamide) (2559, Tocris) were each dissolved in DMSO at 10 mM. At 10 *μ*M of each drug, final DMSO concentrations on the cells were ≤ 0.1%. G-418 (A1720, Sigma-Aldrich) was dissolved at 100 mg/mL in sterile water.

### 2.2 Cell culture, stable transfection, luciferase assay, and adenoviral infection

A549 human pulmonary type II epithelial cells (American Type Culture Collection; ATCC CCL-185; RRID: CVCL_0023) were cultured in Dulbecco’s modified Eagle’s medium supplemented with 10% fetal bovine serum and 2 mM l-glutamine (all Thermo Fisher Scientific). After initial culture and expansion, A549 cells were stored in liquid N_2_ at passage 85–87. Cells were then grown for experiments in continuous culture up to passage ∼100 prior to being discarded. Cells were periodically checked for mycoplasma contamination and tested negative. The day prior to all experiments, cells were placed in serum free medium and this was further changed to fresh serum free medium prior to commencing experimental treatments. Luciferase reporter plasmids were transfected into A549 cells using Lipofectamine 2000 (Invitrogen) diluted in Opti-MEM (Thermo Fisher Scientific) prior to the commencement of selection using G-418 at 700 *μ*g/mL. Following growth of G-418-resistant cells over 2–3 weeks, foci, representing 100s–1000s of integration events, were passaged and the pooled cells cultured in the presence of G-418.^30^ Cells to be used for experiments were plated without G-418 and grown until confluent. Reporter cells were harvested 6 hours after stimulation for luciferase assay using the Firefly Luciferase Assay Kit 2.0 (30085, Biotium Inc). A549 cells harboring the NF-*κ*B reporter (6*κ*Btk.neo) were as previously described.^31^ For adenoviral-mediated transduction, A549 cells were grown to ∼70% confluence prior to infection with a multiplicity of infection (MOI) 25 of a GFP-expression adenovirus, Ad5-GFP, as a control, or adenovirus expressing inhibitor of *κ*B (I*κ*B) *α* with an N-terminal deletion, Ad5-I*κ*B*α*ΔN, to inhibit NF-*κ*B, in serum-containing medium. At this MOI >95% of the cells express the overexpression construct and there is near complete inhibition of IL-1*β*- or TNF*α*-induced NF-*κ*B activity.^32^, ^33^, ^34^ After 24 hours, cells were serum-starved before treatments.

### 2.3 Chromatin immunoprecipitation polymerase chain reaction and sequencing

Chromatin immunoprecipitation (ChIP) was performed as previously described.^35^ After cell treatments, formaldehyde (PI28906, Thermo Fisher Scientific) was added to a final concentration of 1% for 10 minutes. To halt crosslinking, cells were incubated with 125 mM glycine at room temperature for 5 minutes prior to washing with ice-cold PBS supplemented with protease inhibitor cocktail (Thermo Fisher Scientific). Following cytoplasmic and then nuclear lysis, lysates were sonicated with 30 high-power bursts in a 30 seconds on-off cycle at 4 °C using a Bioruptor (Diagenode). Antibodies: GR, GR-356,^36^ RELA (NF-*κ*B p65, D14E12, #8242, Cell Signaling Technologies); RNA polymerase 2 (RNAP2) (Rpb1 NTD D8L4Y, #14958, Cell Signaling Technologies) used for ChIP were preincubated with protein G magnetic Dynabeads (10004D, Thermo Fisher Scientific) overnight at 4 °C and beads were washed as previously described. Nuclear lysates were then incubated with the antibody-coated beads overnight prior to extensive washing. Following reversal of crosslinks, DNA was purified with a ChIP DNA Clean & Concentrator kit (D5205, Zymo Research). Fast SYBR Green Master Mix (Thermo Fisher Scientific) was used in standard quantitative polymerase chain reaction (qPCR) reactions to detect DNA regions of interest. Polymerase chain reaction (PCR) primers were designed to amplify regions (R0–R8) showing RELA and/or GR binding in the *BIRC3* and *BIRC2* loci. Relative occupancy at each region was calculated as ΔΔ threshold cycle (C_T_) after normalization to the geometric mean of the threshold cycle values for 3 negative control regions (Gene symbols: OLIG3, MYOD1, and MYOG). Forward (F) and reverse (R) primers (5ʹ-3ʹ) used for ChIP-qPCR were: MYOD1 (F) TGC AGG AGA TGA AAT ACT AAG CAA GTA, (R) AGA TTG GAA ACT GAG GAC TTT AGT TAG AG; MYOG1 (F) CCA ATG AGA CTG AGT GGG TTT TC, (R) TCA CCA GAG AAG ACT GCT TTG C; OLIG3(F) GGC AAG GAC AGA GAC AAT CAT A, (R) CTC TGT GTT CTC GCT TTG GA; R0 (F) ACT TTG GGA GGA CAC AGT GT, (R) CCC ACC ATG CCC AGT TAA TG; R1 (F) GCT GCC ACC TCA CTG TTT G, (R) AAG GGA AGC CAG GAC AGA AT; R2 (F) CTC TGT ACT CCG CGT ACC CT, (R) TGC ATC TCA TCA GGG CAT CA; R3 (F) GCA CTT ATT CAA GGC TGG TGT, (R) TCT CTG GAG GGA ATT TCG CA; R4 (F) AAT GCC GCG AAG ATA TGC CA, (R) CTC CCA GTG GTT TGC ATG TG; R6 (F) ACA ATA GTG CCA GTT CAA TGA CA, (R) GAT CTA GCA ACA CTG GGC CT; R5 (F) CTG TTT TAG TCG CCA CGC AG, (R) CCC ACG TGA TAA AAA CCC ACA C; R7 (F) TTC TGT GAC TGA CTG GCA GG, (R) CGC TCT TTG CCC GTT GAA TC; R8 (F) CCC TTG GGT GCA GAG AAC TG, (R) AGA ACC AGG TAA AGT GCC ACC. Alternatively, ChIP DNA samples from 2 independent experiments were submitted to the Centre for Health Genomics and Informatics, University of Calgary, for sequencing. Following library preparation (NEB Ultra II kit), samples were subjected to 100-cycle paired-end sequencing (2 × 50 bp) on Illumina NovaSeq 6000 using NovaSeq SP kit v1.5. Demultiplexing was performed using bcl2fastq conversion software (v2.18.0.12) and read quality was assessed using *FastQC* (v0.10.1). Good-quality reads were mapped to GRCh38 reference genome using bowtie2 (v2.4.4) and the resultant BAM files were merged for each treatment and converted to BigWig format prior to uploading to the University of California, Santa Cruz (UCSC) Genome Browser for visualization.

### 2.4 Generation of luciferase reporter constructs

Primers for PCR were designed to flank each region of interest (R0–R8) within ∼100 kb around the *BIRC3* and *BIRC2* loci on chromosome 11. Forward (F) and reverse (R) primers (5ʹ-3ʹ) used were: R0 (F) ACC GTT CAG TGC AGG CTA TG, (R) AGG TCA ACT TTT CCC ACC GT; R1 (F) GTT CGA GCT TCT TGG CTA GTT, (R) GCA TTT CCT CCC TCT CTT TCT T; R2 (F) CTG AGT CCC ACC CCA AAT GA, (R) CTG AGG ACA CTG GGC ATC AT; R3 (F) AAG CCA CCA TTT ATG AGG GGT A, (R) TAC TGT CAG TGT ATG AGC AAG G; R4 (F) GGT GGA GAA CAG GGC ATA TT, (R) ATT GAG CAC CTG GAA CCT ATC; R5 (F) ACA ATA GTG CCA GTT CAA TGA CA, (R) CTG GGC CTA TAG TCC TGC AG; R7 (F) ACC TTC CGA GAA ATA GTG ACT TG, (R) ATA CGT CTG CCT GTG TAT TGG; R8 (F) AAA AGG GGT TTG GGA AGG CA, (R) AGT TGA GAG GAA GGC ATG TAT C. Each region was amplified using Platinum SuperFi PCR Master Mix (Thermo Fisher Scientific) and then introduced into linearized and topoisomerase I activated pCR Blunt II TOPO vector (Thermo Fisher Scientific). DNA fragments were subsequently subcloned using *KpnI* and *XhoI* (both New England Biolabs) into the basal luciferase reporter, pGL3.TATA.neo. This plasmid was previously generated from pGL3basic (Promega) to include a neomycin gene expression cassette and a minimal *β*-globin promoter containing a modified TATA box.^30^ Deletion of putative glucocorticoid response element (GRE), or RELA, binding motifs was performed using the Q5 Site-Directed Mutagenesis Kit (New England Biolabs). Forward (F) and reverse (R) primers (5ʹ-3ʹ) used for mutagenesis of each region were: R2ΔAR (F) TGC CCC ACC CCA AGC TGG, (R) GTA CGT ACA GTT CCA TTA CTG GCC ATC; R4ΔRELA1 (F) GAG TGG GTT TGC CAG GCC, (R) ATG ACC CAA AAG CAT GAC TCT TAA C; R4ΔRELA2 (F) CTA AGT CCT AAA AGG AAA GC, (R) GCG GTA ATA ACC ACA CAC; R4ΔRELA3 (F) AGT GCA CAT GCA AA CCA C, (R) TTT TAG GAC TTA GGG GAA C; R5ΔGRE (F) GTG TCA GGG AAT CAA AGA G, (R) GTA GTC TCC ACT AAC AGT AAC; R7ΔRELA1 (F) CGG CTC CCT AAT TAA GTG, (R) AGA CCC TGA ACC ATT TCC; R7ΔRELA2 (F) AGA GAG AAG CAA GCA TCG, (R) AGG GGC AAA TAT TTA AAA AAC; R8ΔGRE (F) AGA GAC CTT TTG ATA CAT GC, (R) TCC TGT TTC TCT GCT GTG; R8ΔRELA (F) TTC CTG ATC CCA CAG CAG, (R) GGA ACC TCC AGT TCT CTG.

### 2.5 Western blot analysis

Western blotting was carried out as previously described.^9^ In brief, cells were lysed with Laemmli buffer containing protease inhibitor cocktail (PI-78439, Thermo Fisher Scientific) and proteins size fractionated by SDS-PAGE. Proteins were transferred to nitrocellulose membranes before blocking with 5% milk diluted with tris-buffered saline with Tween (TBS-T) and then incubated with primary antibodies against I*κ*B*α* (sc-371), GFP (2555S, CST), or glyceraldehyde-3-phosphate dehydrogenase (MCA4739, Bio-Rad) overnight at 4 °C. Membranes were washed with TBS-T then incubated with either rabbit or mouse horseradish peroxidase-conjugated secondary immunoglobulin (Jackson Immuno Research) at room temperature. Membranes were further washed with TBS-T prior to detection of immune complexes by enhanced chemiluminescence (Bio-Rad). Images were acquired using a ChemiDoc Touch imaging system (Bio-Rad) with densitometric analysis and image representation performed using ImageLab software (Bio-Rad).

### 2.6 RNA extraction and qPCR analysis of mRNA expression

Following treatments, total RNA was extracted and 500 ng used for cDNA synthesis according to standard procedures.^34^ After 1:5 dilution, cDNA (2.5 *μ*L) was analyzed by qPCR (StepOnePlus, Applied Biosciences or QuantStudio3, Thermo Fisher Scientific) with Fast SYBR Green Master Mix (4385618, Thermo Fisher Scientific). Amplification conditions and primers specific for BIRC3, BIRC2, and glyceraldehyde-3-phosphate dehydrogenase mRNAs were as described.^9^ In each case, relative cDNA concentrations were obtained from standard curves generated by serial dilution of a stimulated cDNA sample that was analyzed in parallel with the experimental samples. Primer specificity was assessed by melt curve analysis where a single peak in the change of fluorescence with temperature was taken to indicate specificity.

### 2.7 RNA sequencing and analysis

A549 cells were either not stimulated or treated with IL-1*β* (1 ng/mL), budesonide (300 nM) or IL-1*β*-plus-budesonide for 1, 2, 6, 12, and 24 hours prior to RNA extraction and sequencing at the Centre for Health Genomics and Informatics, University of Calgary. Libraries were prepared from 4 independent experiments using the TruSeq Stranded mRNA Library Prep kits (Illumina) with the poly(A) mRNA magnetic isolation module as specified by the manufacturer. D1000 Screen Tape assays using the Agilent 2200 TapeStation system was used for library validation and quantification was performed by the Kapa qPCR Library Quantification kit. After pooling, libraries were sequenced to ∼20 million reads per sample across 4 consecutive 75 cycle high-throughput sequencing kits using a NextSeq 500 (Illumina). Demultiplexing was performed using bcl2fastq conversion software. Read quality was assessed by FastQC (Illumina) and kallisto, with 100 bootstraps/sample, was used to map good-quality reads to the GRCh38/hg38 reference human transcriptome.^37^ The sleuth package in R was used for normalization and differential gene expression analysis.^38^ Low abundance transcripts (<5 estimated counts in at least 90% of all samples) were filtered out prior to using the Wald test for fold change (*b* value) and false discovery rate determination for each treatment compared with control (no stimulation) at each time. Differentially expressed genes (DEGs) were taken where false discovery rate-adjusted *P* value (*q*) ≤ .05 and log_2_(fold) ≥1 or ≤−1. Data files were deposited with the National Center for Biotechnology Information Gene Expression Omnibus under accession number GSE295743.

### 2.8 Public domain data

Access to Genome Browser sessions for the GR, RELA and RNAP2 ChIP sequencing (seq) and global run-on (GRO)-seq data in BEAS-2B cells was provided by Dr Anthony Gerber (National Jewish Health/University of Colorado) as described.^36^^,^^39^ Micro-C data, obtained from H1-human embryonic stem cells (H1-hESC) and human foreskin fibroblasts (HFFc6) cells,^40^ were accessed and visualized using UCSC Genome Browser, and Hi-C data, from A549 cells,^41^ were accessed through ENCODE project website (encodeproject.org) and visualized using JuiceBox (Juicebox.js).^42^

### 2.9 Data presentation, statistical analyses, and visualization

Data were assumed to be normally distributed and, unless otherwise indicated, are presented as means ± SD with scatterplots showing individual values. With the exception of pEC_50_ values, numerical values in tables and text are provided to one decimal place, or where appropriate 2 significant figures of accuracy. All pEC_50_ and pA_2_ values are provided to 2 decimal places. Multiple comparisons were made using one-way ANOVA with Tukey’s or Dunnett’s post hoc tests, as appropriate. Comparisons between 2 groups were performed by 2-tailed, paired Student’s *t* test. The number of independent experiments (*N*) is indicated in each graph and, being exploratory, sample sizes were not prespecified. To test for greater than simple additivity, the sum of the effects (ie, fold − 1) of each treatment (IL-1*β*, or glucocorticoid alone) (sum) was compared with the effect of cotreatment (comb). GraphPad Prism software (version 11; Dotmatics) and/or the R package “*ggplot2*” was used for graph plotting and statistical analyses. Heat maps were generated using the R package “*pheatmap*.” Concentration-response relationships were fitted using the 4-parameter model that assumed symmetric variance or using the Gaddum-Schild model of competitive antagonism, as described previously.^43^ Variance in pEC_50_, *E*_Max_, or pA_2_ are provided by Prism as mean ±SE, whereas all curves are plotted as means ±SD (but without scatterplots). Because all analyses were exploratory and were not designed to test prespecified statistical null hypotheses, stated *P* values should be viewed as being descriptive rather than as hypothesis testing.

ChIP-seq data, expressed as normalized read count/million, were visualized using the UCSC Genome Browser (https://genome.ucsc.edu) or the Bioconductor package “*Gviz”* in R. Genomic regions, GRO-seq, and micro-C data were visualized using the UCSC Genome Browser. To maintain consistency between figures, the default UCSC Genome Browser annotation of (+) and (−) strands was adopted for describing the orientation of genomic regions. Hi-C chromatin interaction matrices were visualized using “Juicebox” webtool (https://aidenlab.org/juicebox).^42^ Sequence analysis of binding sites to identify GRE, RELA-binding motifs or androgen receptor (AR)–binding motifs sequence was performed using JASPAR (http://jaspar.genereg.net).^44^ Gene ontology (GO) analyses were performed using the functional annotation tool within the Database for Annotation, Visualization, and Integrated Discovery.^45^ GO categories for extraction were limited to biological process (GOTERM_BP_DIRECT) and Kyoto Encyclopedia of genes and genomes (KEGG) pathway terms. The Benjamini correction for multiple testing of enrichment *P* values (*P*_*B*_) was used and *P*_*B*_ ≤ .05 was taken to highlight enriched terms.

## 3 Results

### 3.1 Recruitment of RELA and GR to the *BIRC3* and *BIRC2* loci

**Fig. 1:**
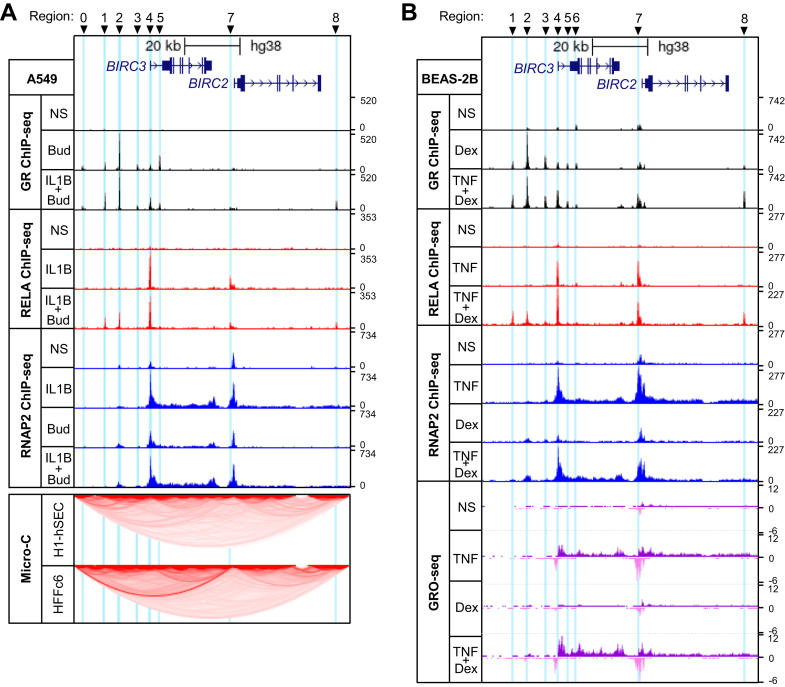
GR, RELA, and RNAP2 ChIP-seq and micro-C analysis of the *BIRC3*/*BIRC2* genomic locus. Genome browser snapshot of ∼100 kb encompassing the *BIRC3* and *BIRC2* loci. Arrow heads within the intronic regions indicate direction of transcription. Highlighted areas (light blue) correspond to GR and/or RELA-binding regions (R0–R8). (A) A549 cells were either not stimulated (NS) or treated with budesonide (Bud; 300 nM), IL-1*β* (1 ng/mL), or both for 1 hour prior to ChIP-seq analysis for GR (black), RELA (red), and RNAP2 (blue). Normalized ChIP-seq traces from 2 independent experiments are shown (upper 10 tracks). Micro-C data from H1-hESC and HFFc6 cells were mapped to the same chromosome location to reveal chromatin structure (lower 2 tracks). (B) GR (black), RELA (red), and RNAP2 (blue) ChIP-seq traces from BEAS-2B cells that were not stimulated or treated for 1 hour with dexamethasone (Dex; 100 nM), TNF*α* (TNF; 20 ng/mL) or both are shown (upper 10 tracks). GRO-seq traces from a similar experiment are shown (lower 4 tracks; positive strand in purple and negative strand in pink). In each case, peak heights reflect normalized read density as visualized using the UCSC Genome Browser.

Prior analyses indicated roles for NF-*κ*B, in particular RELA, as well as GR in the regulation of BIRC3 expression by IL-1*β* and glucocorticoid in A549 cells.^8^^,^^9^ Conversely, BIRC2 mRNA expression was more modestly induced by IL-1*β* and this effect was reduced by glucocorticoids. To investigate roles for RELA and GR in the regulation of BIRC3 and BIRC2 expression, ChIP sequencing was performed for GR and RELA in A549 cells following treatment for 1 hour with maximally effective concentrations of IL-1*β* (1 ng/mL), budesonide (300 nM) or the combination of IL-1*β*-plus-budesonide. Displaying these data in the genome browser revealed a striking resemblance to data generated from BEAS-2B cells in which regions R0–R8 were identified as binding GR and/or RELA following treatment with TNF*α* or/and dexamethasone (Fig. 1).^39^

In A549 and BEAS-2B cells, IL-1*β* or TNF*α*, respectively, induced prominent RELA recruitment, referred to as RELA-binding regions (RBRs), to 2 DNA regions (R4 and R7) that were situated ∼120 bp and ∼1 kb 5′ to the *BIRC3* and *BIRC2* transcription start sites (TSSs), respectively (Fig. 1, A and B). Similarly, budesonide, in A549 cells, and dexamethasone, in BEAS-2B cells, produced a strong GR-binding region (GBR) at R2, located ∼11 kb upstream of the *BIRC3* TSS (Fig. 1A). Multiple, but lesser, GBRs were apparent at R1, R3, R4, and R5 (Fig. 1A). The modest, but unregulated, GBR observed at R6 in BEAS-2B cells was not discernable in A549 cells, whereas a minor GBR, detected at R0 in the A549 cells, was not detected in BEAS-2B cells. With IL-1*β*-plus-budesonide cotreatment, RELA recruitment to the R4 RBR was largely unaltered, whereas at R1 and R2 new RBRs were now evident where with IL-1*β* alone there was previously no RBR (Fig. 1A). Similar effects, with the gain of new RBRs at R1 and R2, also occurred in BEAS-2B cells treated with TNF*α*-plus-dexamethasone (Fig. 1B). Appearance of a new RBR was also observed at R8 on cytokine-plus-glucocorticoid cotreatment of both A549 and BEAS-2B cells. In A549 cells, the IL-1*β*-induced RBR at R7, just proximal to the *BIRC2* TSS, showed a modest reduction in intensity with IL-1*β*-plus-budesonide, whereas little or no change to RELA recruitment was apparent in BEAS-2B cells with TNF*α*-plus-dexamethasone compared with TNF*α* alone. At this R7 region, GR recruitment was absent with budesonide or IL-1*β*-plus-budesonide in A549 cells, whereas in BEAS-2B cells GR recruitment appeared unchanged with dexamethasone alone, but was modestly increased by TNF*α*-plus-dexamethasone. On cotreatment there were also increases in GR recruitment at the R1, R4, and R8 GBRs, but no change at R2 or the more minor GBRs (R0 and R3) in either cell line. In A549 cells, GR recruitment to the R5 GBR, which did not show IL-1*β*-plus-budesonide-induced recruitment of RELA, was reduced by cotreatment. In BEAS-2B cells this reduction in GR at R5 was not apparent with TNF*α*-plus-dexamethasone cotreatment.

Taken together, GR and RELA recruitment was clear at various DNA regions in the *BIRC3* and *BIRC2* loci in A549 cells with budesonide or IL-1*β*, respectively. On cotreatment, GR and RELA colocalized to: R1 and R2, both of which gained new RBRs; R4, where the GBR was increased; and R8, which showed increased recruitment of both GR and RELA (Fig. 1A). Conversely, in A549 cells there was no RELA recruitment observed at R5 and this region showed reduced GR with combination treatment. Finally, recruitment of RELA to R7 associated with very low levels of GR in the combination treatment and RELA binding was reduced in A549 cells compared with IL-1*β* treatment. That these effects were virtually identical in BEAS-2B cells treated with TNF*α* and/or dexamethasone supports common mechanisms of action (Fig. 1B).

### 3.2 RNAP2 ChIP and GRO-seq indicate transcriptional activation

In untreated A549 and BEAS-2B cells, RNAP2 was present at low levels around both the *BIRC3* and *BIRC2* TSSs (Fig. 1). In both cell lines, RNAP2 presence was increased by glucocorticoid at the *BIRC3* TSS, as well as at the main GBR-containing region, R2. Little or no glucocorticoid-dependent changes in RNAP2 were evident at the *BIRC2* TSS or at R7. In A549 cells treated with IL-1*β* and BEAS-2B cells treated with TNF*α*, RNAP2 recruitment was increased at R4 and in the vicinity of both TSSs. This was paralleled by increased RNAP2 along both the *BIRC3* and *BIRC2* gene bodies. Such data support increased gene transcription and are therefore consistent with the previously reported increases in expression for both genes.^9^ In the context of IL-1*β*-, or TNF*α*-, plus-glucocorticoid, RNAP2 presence at R4 or the *BIRC3* TSS did not change relative to that induced by either cytokine alone. Glucocorticoid-induced RNAP2 detected at, or around, R2 was unchanged with the combination in A549 cells, but appeared to be modestly increased with TNF*α*-plus-dexamethasone in BEAS-2B cells. In both cell lines, cytokine-plus-glucocorticoid induced RNAP2 levels associated with the *BIRC3* gene body was similar to that for cytokine stimulation alone. However, in BEAS-2B cells, the combination treatment modestly reduced RNAP2 at the *BIRC2* TSS and R7 compared with cytokine alone, but this effect was less evident in A549 cells (Fig. 1). Cytokine-induced RNAP2 along the main body of the *BIRC2* gene was also reduced in BEAS-2B cells by combination treatment.

Cytokine-induced transcriptional activation of the *BIRC3* and *BIRC2* genes was strongly supported by GRO-seq data obtained from BEAS-2B cells treated with TNF*α*, dexamethasone or TNF*α*-plus-dexamethasone.^36^ These data reveal bidirectional nascent transcripts, an established marker of active enhancers,^46^^,^^47^ induced by TNF*α* from R4 and R7 (Fig. 1B). Furthermore, this effect extended through the gene bodies of both *BIRC3* and *BIRC2* to confirm transcriptional activation of both genes. With dexamethasone treatment, run-on transcripts from R7 and along the *BIRC2* gene were at similar levels to unstimulated. This indicates a basal level of *BIRC2* gene transcription that was unaffected by glucocorticoid. At *BIRC3*, very low levels of run-on transcripts occurred in unstimulated cells (Fig. 1B). This was largely unaffected by dexamethasone alone, but with TNF*α*-plus-dexamethasone, modestly enhanced levels of run-on transcripts were observed at R2, R3, R4/TSS, as well as along the whole *BIRC3* gene compared with each treatment alone. At R7 and the *BIRC2* TSS, levels of run-on transcripts produced by TNF*α*-plus-dexamethasone were similar to that for TNF*α* alone, whereas the run-on signal along the *BIRC2* gene body was modestly reduced (Fig. 1B).

Taken together, the RNAP2- and GRO-seq data are consistent with basal transcription of *BIRC2*, and to a lesser extent *BIRC3*, which leads to the observed mRNA and protein expression of both genes in untreated cells.^9^ These data also support modest increases in nascent *BIRC3* transcripts leading to the elevated mRNA and protein that was observed following glucocorticoid treatment. Likewise, inflammatory cytokines increase the expression of both BIRC3 and BIRC2 mRNAs,^9^ effects that are consistent with the inducible presence of RNAP2 and GRO-seq transcripts at each TSS and along the gene bodies of both *BIRC3* and *BIRC2*. With the combination of cytokines and glucocorticoid, the presence of RNAP2 and GRO-seq transcripts at *BIRC3* was maintained or enhanced relative to cytokine alone, whereas RNAP2 and run-on transcripts at *BIRC2* were reduced. In each case, these data are consistent with established effects on gene expression for both genes.^9^ Furthermore, the presence of RNAP2 and bidirectional transcripts induced by glucocorticoid at R2 and by cytokines at R4 and R7 supports a role for inducible enhancers at each region. Finally, micro-C data from untreated pluripotent H1-hESCs and HFFc6 were used to identify possible DNA looping events.^40^ Aligning the micro-C map to the ∼100 kb spanning the *BIRC3* and *BIRC2* loci revealed DNA looping between the *BIRC3* R4/TSS region and multiple regions that included R0–R3 (Fig. 1A, lower panels). This region also appears to loop to R7 and the *BIRC2* TSS, which indicates further interaction events that connect upstream to R0, as well as downstream to R8. These data provide a rationale to examine transcriptional control at each region.

### 3.3 ChIP-qPCR validation of RBRs and GBRs at the *BIRC3*/*BIRC2* locus

**Fig. 2:**
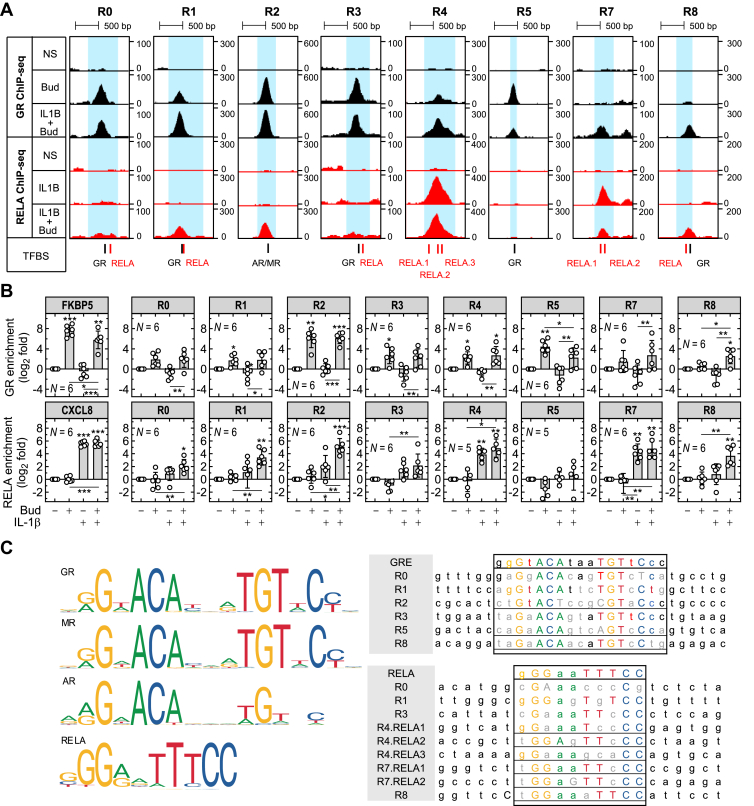
Differential recruitment of GR and RELA to regions in the *BIRC3*/*BIRC2* locus. (A) Genome browser snapshots of ChIP-seq traces for each genomic region (R0–R8) showing GR (black) and RELA (red) binding in A549 cells that were either not stimulated (NS), treated with budesonide, (Bud; 300 nM), IL-1*β* (1 ng/mL), or both. GRE- (black boxes) and RELA motifs (red boxes), as predicted from the JASPAR CORE database (score ≥ 300), are indicated. For R2, the AR/MR motif is indicated. Normalized read density is visualized using the UCSC Genome Browser. (B) ChIP-qPCR for GR and RELA was performed in A549 cells that were either not stimulated or stimulated with budesonide, (Bud; 300 nM), IL-1*β* (1 ng/mL), or both for 1 hour. qPCR data were normalized to the geometric mean of 3 control regions that are not occupied by GR or RELA. Normalized data, from *N* independent experiments (each performed with 2 technical replicates), were plotted as log_2_ fold enrichment from not stimulated as mean ±SD. Significance, using normalized data, was tested with one-way ANOVA with a Tukey’s post hoc test. ∗*P* ≤ .05, ∗∗*P* ≤ .01, ∗∗∗*P* ≤ .001 indicates significance to no stimulation, or as indicated. (C) Consensus position weight matrix (PWM) for GR, MR, AR, or RELA binding motifs in human cells, as identified by the JASPAR CORE database. Sequences for the GREs and/or RELA motifs within each GBR or RBR of interest are shown. The black box indicates the sequence region of interest where colored uppercase letters indicate ≥0.75 match and colored lowercase letters indicate ≥0.5/<0.75 match to the consensus GRE or RELA motif. Black lowercase letters represent the highest called letter and gray indicates no match to the consensus. The underlined sequences were deleted by site-directed mutagenesis.

To more rigorously explore changes in GR and RELA recruitment to regions R0–R8 (Fig. 2A), additional ChIP-qPCR experiments were performed in A549 cells treated with IL-1*β*, budesonide or the combination for 1 hour. Primers were designed to each GBR/RBR (R0–R8) and the presence of ChIP DNA was assessed by qPCR. Initial ChIP-qPCR focused on a previously validated GBR in the *FKBP5* locus,^35^ plus an RBR just 5′ of the *CXCL8* TSS. In A549 cells, these genes are glucocorticoid-induced and IL-1*β*-induced, respectively,^25^^,^^48^ and showed strong GR or RELA recruitment in the current ChIP sequencing data (Supplemental Fig. 1). Thus, ChIP-qPCR confirmed robustly induced recruitment (240 ± 150-fold) of GR to *FKBP5* by budesonide, an effect that was reduced (*P* ≤ .05) by IL-1*β* (Fig. 2B). Similarly, IL-1*β*-induced RELA was strongly recruited (48 ± 11-fold) to *CXCL8*, an effect that was unchanged with IL-1*β*-plus-budesonide (Fig. 2B). These data provide general validation of the ChIP-seq experiments and allow more detailed analysis of GR and RELA recruitment to the *BIRC3* and *BIRC2* loci.

Following 1 hour of budesonide, ChIP-qPCR confirmed increased GR occupancy at R1, R2, R3, R4, and R5 (all at least *P* ≤ .05) with a modest, but statistically not significant level of GR binding apparent at R0 and R8 (Fig. 2B). Because GR occupancy at R2 showed the greatest overall enrichment (Fig. 2), these data were broadly consistent with the ChIP sequencing data (Fig. 1). GR occupancy at R2 and R4 also increased (both *P* ≤ .05) by IL-1*β*-plus-budesonide, whereas significance was lost at R1, R3, and R5 (Fig. 2B). At R2 and R4, IL-1*β*-plus-budesonide-induced GR binding was similar to that for budesonide alone, whereas at R8 GR presence was significantly (*P* ≤ .05) more than with budesonide alone (Fig. 2B). At R5, budesonide-induced binding of GR was significantly (*P* ≤ .05) decreased with IL-1*β*-plus-budesonide cotreatment.

In cells treated with IL-1*β*, ChIP-qPCR confirmed increases (both *P* ≤ .01) in RELA occupancy at both R4 and R7, the 2 main RBRs identified by ChIP sequencing, but not at other regions (Fig. 2). In cells treated with IL-1*β*-plus-budesonide, there were also statistically significant increases in RELA occupancy at R0, R1, R2, R4, R7, and R8 compared with unstimulated cells. Furthermore, at R2, the increase in RELA binding with IL-1*β*-plus-budesonide was significantly greater than for IL-1*β* treatment alone (Fig. 2B).

The above data confirm overall reliability of the GR and RELA ChIP sequencing data and support consistent region-specific changes in GR and RELA recruitment upon IL-1*β*-plus-glucocorticoid treatment relative to each monotreatment. Thus, the glucocorticoid-induced GBRs at R0, R1, and R2 each recruited GR. These effects were largely unaffected by IL-1*β*-plus-glucocorticoid, but were associated with gains in RELA on combination treatment. The much weaker R3 GBR was also relatively unaffected by IL-1*β*, as cotreatment and recruitment of RELA were modest and not significant. This contrasts with the R5 GBR, which was induced by glucocorticoid, but was significantly reduced on combination treatment and did not materially recruit RELA. Turning to R4 and R7, these regions were principally IL-1*β*-induced RBRs that recruited RELA in a manner that was relatively unaffected by glucocorticoid as cotreatment. However, both these regions also showed evidence, significant (*P* ≤ .05) for R4, of GR occupancy with budesonide alone, or as cotreatment. Finally, the R8 region revealed very low glucocorticoid-induced recruitment of GR that was markedly enhanced upon cotreatment, whereas RELA occupancy to this region was only significantly induced (*P* ≤ .01) with IL-1*β*-plus-budesonide.

### 3.4 GR and NF-κB motifs at GBRs and RBRs within the *BIRC3*/*BIRC2* locus

Motif analysis was performed using the JASPAR CORE database on the depicted regions around each of the R0–R8 GBR/RBR peaks (Fig. 2A).^44^ All RELA or GR binding motifs, that is, GREs, with similarity scores ≥300, that is, corresponding to *P* ≤ 10^−3^, and which therefore represent significant matching to the NF-*κ*B (RELA) and GRE consensus sequences, respectively, are shown (Fig. 2C). A single GRE motif, at or close to the peak of each GBR, was identified at R0, R1, R3, R5, and R8 (Fig. 2A). At R2, the GBR showing the greatest GR enrichment, no consensus GR motif was identified, even when the stringency was lowered to a score of ≥100 (ie, *P* ≤ 10^−1^). However, inclusion of motifs for the AR and mineralocorticoid receptor (MR) in the search revealed a strong AR/MR motif (score ≥300) (Fig. 2A), which given the known and clear homology with the GR motif (Fig. 2C),^44^^,^^49^ raises the prospect of GRE-like behavior. Similarly, a RELA binding motif was identified in each of R0, R1, R3, and R8. R4 revealed 3 RELA motifs within the RBR ChIP sequencing peak and R7 revealed 2 RELA motifs (Fig. 2A).

### 3.5 Transcriptional activity of GR and RELA-binding regions from the *BIRC3*/*BIRC2* locus

**Fig. 3:**
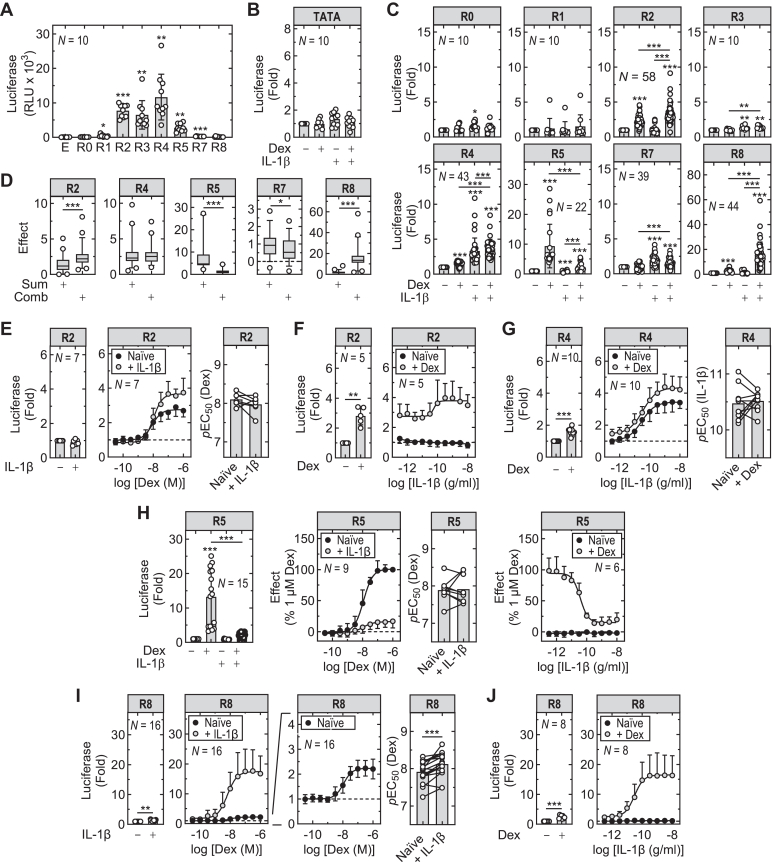
Transcriptional activity of GR- and RELA-binding regions at the *BIRC3*/*BIRC2* locus. The regions R0–R8, as highlighted in Fig. 2A, were cloned into a TATA-containing luciferase vector prior to stable transfection into A549 cells. (A) Basal reporter activity of the empty vector (E) and the R0–R8 reporter constructs was assessed and plotted as relative light units (RLU) ± SD. (B and C) Cells harboring the empty TATA containing vector, or the R0–R8 reporter constructs were either not stimulated or stimulated with dexamethasone (Dex; 1 *μ*M), IL-1*β* (1 ng/mL), or both. (D) The sum of the effects (ie, fold – 1) for each treatment (*sum*) and the effect (fold – 1) of the combination treatment (*comb*) are shown. (E) The R2 reporter was either not stimulated or treated with the indicated concentrations of dexamethasone each in the absence or presence of IL-1*β* (1 ng/mL). Using paired data from each individual experiment, curve fitting was performed for each treatment (naïve, +IL-1*β*) and individual pEC_50_ values plotted (right panel). (F) The R2 reporter was either not stimulated or treated with the indicated concentrations of IL-1*β* each in the absence or presence of dexamethasone (1 *μ*M). (G) The R4 reporter was treated and graphs plotted as in (E). (H) The R5 reporter was treated as in (E and F). Owing to variability in fold values between reporter batches, data for dexamethasone (1 *μ*M), IL-1*β* (1 ng/mL) and both together were, for clarity, plotted as fold of no stimulation (left panel). Data for each concentration series (dexamethasone or IL-1*β*) were then expressed as % effect of dexamethasone at 1 *μ*M where no stimulation = 0 and 1 *μ*M dexamethasone = 100. pEC_50_ values were obtained as in (E). (I) The R8 reporter was treated and graphs plotted as in (E). Note, expansion of *x*-axis shows the response to dexamethasone alone. (J) The R8 reporter was treated and graphs plotted as in (F). All cells were harvested 6 hours after treatments for luciferase assay. Unless otherwise indicated, data from *N* independent experiments were plotted as fold of no stimulation ± SD. Data are shown as bar graphs overlaid with scatter plots or as fitted curves with means ± SD. Significance in: A was tested by one-way ANOVA with a Dunnett’s post hoc test; B, C, and H (left panel) was tested by one-way ANOVA with a Tukey’s post hoc test; in D–F paired *t* tests were used. ∗*P* ≤ .05, ∗∗*P* ≤ .01, ∗∗∗*P* ≤ .001 indicates significance to not stimulated, or as indicated.

To evaluate the capacity for regions R0–R8 to promote transcription, segments of DNA (highlighted in Fig. 2A), were cloned into a TATA-containing luciferase reporter (pGL3basic.TATA.neo) prior to transfection into A549 cells and generation of stable reporter lines. These were treated with IL-1*β*, dexamethasone, or both for 6 hours prior to performing luciferase activity assays to assess transcriptional activity. Without stimulation, the reporter lines R2, R3, and R4 all showed basal transcriptional activity that was 100–200-times higher than the parent TATA-containing reporter (Fig. 3A). The R0, R1, R7, and R8 reporters all showed lower (1–10-fold) activity relative to the empty vector and R5 reporter revealed intermediate (45-fold) activity.

The TATA reporter, along with R0, R1, and R3, were largely unaffected by IL-1*β*, dexamethasone or IL-1*β*-plus-dexamethasone, albeit significant inductions of 1.6 ± 0.5-fold (*P* ≤ .05) for R0 and 1.4 ± 0.3-fold (*P* ≤ .01) for R3 were observed with IL-1*β* (Fig. 3, B and C). R3 also showed a similar effect (*P* ≤ .01) with IL-1*β*-plus-dexamethasone. Dexamethasone resulted in 2.4 ± 0.8, 1.5 ± 0.3, 9.4 ± 7.3, and 2.6 ± 0.9-fold increases (all *P* ≤ .001) in activity for R2, R4, R5, and R8, respectively, whereas IL-1*β* produced 3.3 ± 1.9, 2.0 ± 0.7, and 1.3 ± 0.7-fold increases in R4, R7, and R8, respectively (Fig. 3C). With IL-1*β*-plus-dexamethasone, R2 activity increased (3.4 ± 1.3; *P* ≤ .001) in a manner that was also greater (*P* ≤ .001) than that observed for dexamethasone alone. Likewise, IL-1*β*-plus-dexamethasone induced R4 activity to 3.7 ± 1.3-fold and this was greater than for dexamethasone (*P* ≤ .001) or IL-1*β* (*P* ≤ .001) alone. Conversely, the 9.4-fold dexamethasone-induced drive at R5 was markedly reduced (*P* ≤ .001), albeit to a still significant 2.4 ± 1.3-fold (*P* ≤ .001), with the combination treatment. With R7 the already modest 2.0-fold IL-1*β*-induced drive was reduced to 1.6 ± 0.6 (*P* ≤ .001). Finally, at R8 the modest effects of dexamethasone and IL-1*β* alone produced a marked 16.5 ± 9.6-fold (*P* ≤ .001) increase when combined. Analyses of the R0–R5, plus R7 and R8 reporters following treatment with dexamethasone and/or TNF*α* at 10 ng/mL, a maximally effective concentration,^9^ resulted in near-identical effects to that observed for IL-1*β* (Supplemental Fig. 2).

As most clearly illustrated at R8, the above data suggest interactions between the glucocorticoid and IL-1*β* acting on a number of these reporters. To formally test this, the sum of the effects, being the sum of each fold – 1 for the 2 treatments, and the effect of dexamethasone-plus-IL-1*β* were compared for R2, R4, R5, R7, and R8 (Fig. 3D). Thus, the sum of the effects of dexamethasone and IL-1*β* was markedly (*P* ≤ .001) less than the effect for the combination indicating greater than, or s*upra*-, additivity for R8. Performing this analysis on the glucocorticoid-inducible, but IL-1*β*-enhanced (Fig. 3C), R2 reporter, also revealed *supra*-additivity (*P* ≤ .001) (Fig. 3D). This contrasts with R4 where the primary IL-1*β*-induced response, combined with the more modest dexamethasone response summated in a simple additive manner (Fig. 3D). Finally, at both R5 and R7, where the response to either dexamethasone or IL-1*β*, respectively, was reduced by the combination treatment (Fig. 3C), the effect of the combination was significantly (*P* ≤ .001 and .05, respectively) less, or *infra*-additive, for each reporter when compared with the sum of the individual effects (Fig. 3D). Given that these outcomes all occurred at maximally effective concentrations of glucocorticoid and IL-1*β*,^9^^,^^30^ positive cooperativity, or synergy, between the glucocorticoid and IL-1*β* may be inferred where *supra*-additivity was shown. Equally, negative cooperativity is suggested by *infra*-additivity.

### 3.6 Reporter sensitivity and effect of cotreatment

To further explore the combinatorial effects of IL-1*β* and glucocorticoid on the different reporters, effects of concentration were tested on the R2, R4, R5, and R8 reporters. R7 was not examined because of its modest inducibility combined with low overall reporter activity, making this construct unsuitable for analysis.

At the R2 reporter, there was no effect of IL-1*β* alone and concentration-dependent increases were shown for dexamethasone (pEC_50_ 8.07) with an *E*_Max_ of 2.8-fold (Fig. 3E; Table 1). This response was enhanced to 3.7-fold in the presence of IL-1*β* without obvious change in the sensitivity to dexamethasone. Similar outcomes were also shown for budesonide, which produced a pEC_50_ of 8.46 that was not significantly altered in the presence of IL-1*β* and at 300 nM produced indistinguishable effects from dexamethasone at 1 *μ*M (Supplemental Fig. 3, A and B; Supplemental Table 1). In the presence of dexamethasone (1 *μ*M), IL-1*β* produced a concentration-dependent (pEC_50_ 10.25) increase in reporter activity from 2.8 ± 0-fold with dexamethasone alone to 4.0 ± 1.1-fold with IL-1*β* at 1 ng/mL, but alone was without effect (Fig. 3F; Table 1).

**Table 1:** Effect of dexamethasone and IL-1*β* on potency and efficacy at the R2, R4, R5, and R8 *BIRC3*/*BIRC2* reporters Data from the concentration responses analyses in Fig. 3 were subjected to 4-parameter curve fitting using GraphPad Prism v11 to produce overall pEC_50_ and E_Max_ values, each with an associated SE.

Reporter	Treatment									
	Dexamethasone					IL-1*β*				
	Naïve		+ IL-1*β*		*N*	Naïve		+ Dexamethasone		*N*
	*E*_Max_ (Fold^a^ ± SE)	pEC_50_ (−log[M] ± SE)	*E*_Max_ (Fold^a^ ± SE)	pEC_50_ (−log[M] ± SE)		*E*_Max_ (Fold^a^ ± SE)	pEC_50_ (−log[g/ mL] ± SE)	*E*_Max_ (Fold^a^ ± SE)	pEC_50_ (−log[g/mL] ± SE)	
R2	2.8 ± 0.1	8.07 ± 0.09	3.7 ± 0.1	7.99 ± 0.08	7	n.r.	n.r.	3.8 ± 0.2	10.25 ± 0.33	5
R4	n.d.	n.d.	n.d.	n.d.		3.5 ± 0.1	10.54 ± 0.10	4.3 ± 0.1	10.46 ± 0.11	10
R5^a^	101% ± 2	7.94 ± 0.04	16.3% ± 1.8	8.00 ± 0.18	9	n.r.	n.r.	15.1% ± 3.1	10.43 ± 0.08	6
R8	2.2 ± 0.0	7.95 ± 0.6	17.5 ± 0.8	8.18 ± 0.10	16	n.r.	n.r.	16.5 ± 1.0	10.51 ± 0.15	8

n.d., not determined; n.r., no significant response.

a R5 reporter data are expressed as % of 1 *μ*M dexamethasone (13.26 ± 2.12 fold).

Dexamethasone at 1 *μ*M showed a ∼1.5-fold increase in R4 reporter activity (Fig. 3C), which, owing to the small effect size, was not subjected to concentration-response analyses. However, testing the effect of IL-1*β* concentration in the absence and presence of 1 *μ*M dexamethasone confirmed a 1.6 ± 0.2-fold effect (*P* ≤ .001) for dexamethasone alone that produced similar increases at all IL-1*β* concentrations (Fig. 3G). These data do not deviate from simple additivity and dexamethasone produced no significant (*P* > .05) change in the potency of IL-1*β* at the R4 reporter (Fig. 3G; Table 1). Budesonide produced similar increases in R4 reporter activity to dexamethasone and modestly reduced the sensitivity to IL-1*β* (naïve pEC_50_ = 10.63, with IL-1*β* pEC_50_ = 10.45) (Supplemental Fig. 3, C and D; Supplemental Table 1).

The strong, albeit variable level with different batches of reporter cells, response to dexamethasone by the R5 reporter, and its inhibition by IL-1*β*, was recapitulated and sensitivity to dexamethasone (pEC_50_ 7.94 ± 0.04) appeared largely unaffected by IL-1*β* (Fig. 3H; Table 1). Budesonide produced both a similar *E*_Max_ to dexamethasone and was similarly repressed by IL-1*β* (Supplemental Fig. 3E). However, in these experiments, IL-1*β* produced a modest but significant (*P* ≤ .01) drop in the pEC_50_ to budesonide (Supplemental Table 1). Although the R5 reporter was unresponsive to IL-1*β* alone (Fig. 3, C and H), inhibition of dexamethasone-induced reporter activity by IL-1*β* occurred with a sensitivity (pEC_50_ = 10.43 ± 0.08) that produced maximal effects in the 0.1–1 ng/mL range (Fig. 3H; Table 1).

At R8, although IL-1*β* alone showed only a very modest 1.2 ± 0.3-fold (*P* ≤ .01) effect, the 2.2-fold *E*_Max_ in response to dexamethasone was increased to 17.5 ± 0.8-fold in the presence of IL-1*β* (Fig. 3I; Table 1), an effect that was recapitulated by budesonide (Supplemental Fig. 3F). Along with increased efficacy, the pEC_50_ for dexamethasone increased (*P* ≤ .001) from 7.95 to 8.18 in the presence of IL-1*β* (Fig. 3I; Table 1), a result that was also apparent for budesonide (Supplemental Fig. 3G). Testing of various IL-1*β* concentrations alone showed no significant effect on the R8 reporter. However, in the presence of dexamethasone (1 *μ*M), or 300 nM budesonide, IL-1*β* produced a robust response (pEC_50_ = 10.51 or 10.47, respectively) resulting in maximal effects from ∼0.1 ng/mL with each glucocorticoid (Fig. 3J; Table 1; Supplemental Fig. 3H; Supplemental Table 1).

### 3.7 Roles of GR and RELA binding motifs in transcriptional activity

**Fig. 4:**
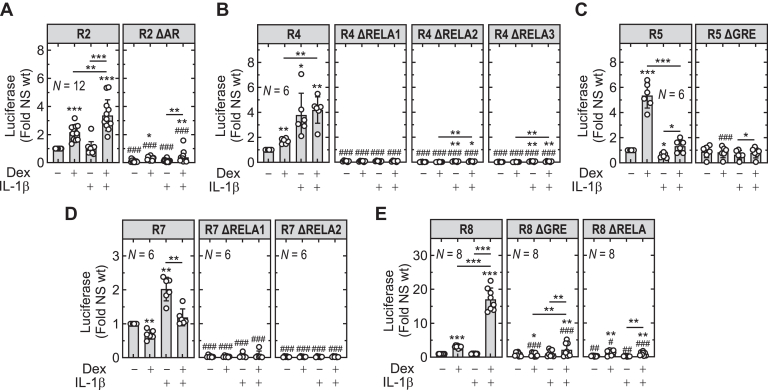
Effect of GRE or RELA motif deletion on R2, R4, R5, R7, and R8 reporter activity. Each GRE or RELA motif within the R2, R4, R5, R7, and R8 reporters, as depicted in Fig. 2, was deleted by site-directed mutagenesis to give the respective ΔAR, ΔGRE, or ΔRELA constructs. Wild-type and mutated constructs were transfected into parallel batches of A549 cells. Stably transfected cells for: (A) R2 and R2ΔAR; (B) R4, R4ΔRELA1, R4ΔRELA2, and R4ΔRELA3; (C) R5 and R5ΔGRE; (D) R7, R7ΔRELA1, and R7ΔRELA2; and, (E) R8, R8ΔGR, and R8ΔRELA were either not stimulated or stimulated with dexamethasone (Dex; 1 *μ*M), IL-1*β* (1 ng/mL), or both. All experiments were performed in parallel on all the constructs for each region. Cells were harvested 6 hours after treatments for luciferase assay. Data from *N* experiments, expressed as fold of untreated relative to the wild-type versions of each construct, were plotted as mean ± SD overlaid with scatter plots. Significance was tested by one-way ANOVA with a Tukey’s post hoc test. For each reporter construct ∗*P* ≤ .05, ∗∗*P* ≤ .01, ∗∗∗*P* ≤ .001 indicates significance to its own not stimulated or as otherwise indicated. ^#^*P* ≤ .05, ^##^*P* ≤ .01, ^###^*P* ≤ .001 indicates significance between each deletion and the wild-type construct for each treatment.

To explore roles for GR and RELA motifs in the transcriptional drive observed from R2, R4, R5, R7, and R8, each putative motif, as identified in Fig. 2C, was deleted and effects on reporter activity assessed. As shown in Fig. 3, the R2 reporter revealed dexamethasone-induced activity that was modestly enhanced by IL-1*β*, even though IL-1*β* alone was without effect (Fig. 4A). Removal of the AR/MR motif substantially reduced basal and induced R2 reporter activity (each *P* ≤ .001) in the context of each treatment. However, despite this, both dexamethasone, and dexamethasone-plus-IL-1*β* (*P* ≤ .05 and .01, respectively) induced R2 activity with fold values that were not significantly different from that for the wild-type reporter (Supplemental Fig. 4A). As with the wild-type reporter, IL-1*β* was without effect. Thus, although the AR/MR motif was critical for R2 reporter drive, this motif alone may not explain inducibility by glucocorticoid.

The R4 reporter was modestly inducible by dexamethasone with a more substantial response to IL-1*β* that combined as simple additivity with both treatments together (Fig. 4B). Deletion of any 1 of the 3 RELA motifs (R4 RELA1–3) profoundly (all *P* ≤ .001) reduced basal and stimulated reporter activity (Fig. 4B). Deletion of R4 RELA1 abrogated the responses to dexamethasone, IL-1*β* and IL-1*β*-plus-dexamethasone (Fig. 4B; Supplemental Fig. 4B). With the R4 RELA2 and R4 RELA3, each deletion resulted in loss (both *P* ≤ .01) of the modest response to dexamethasone without apparent effect on inducibility by IL-1*β* (Fig. 4B; Supplemental Fig. 4B). Notably, neither of these 2 RELA deletions showed evidence for further enhancement of the IL-1*β* response in the combination treatment. Thus, although RELA1 was essential for inducibility by IL-1*β* and all 3 RELA motifs were necessary for overall reporter drive, each of these 3 RELA motifs appeared important in mediating dexamethasone-responsiveness.

As described in Fig. 3, the R5 reporter, representing a GBR that did not recruit RELA (Fig. 2), was induced by dexamethasone in a manner that was strongly repressed by IL-1*β* in the combination treatment, whereas IL-1*β* alone modestly reduced basal reporter activity (Fig. 4C; Supplemental Fig. 4C). Deletion of the single GRE present in R5 had no effect on basal reporter activity, but completely prevented dexamethasone-induced luciferase activity (Fig. 4C; Supplemental Fig. 4C). The ability of IL-1*β* to repress basal activity of this reporter was also prevented. Thus, the GRE motif in R5 behaved in a classical manner and was necessary for glucocorticoid-induced transcription.

Region R7 shows 2 putative RELA motifs upstream of the *BIRC2* TSS and revealed a modest 2.0-fold transcriptional drive induced by IL-1*β* (Figs. 2A and 3C). Deletion of each of R7 RELA1 and R7 RELA2 profoundly reduced basal and stimulated reporter activity (all *P* ≤ .001) (Fig. 4D; Supplemental Fig. 4D). Furthermore, neither deletion construct was inducible by IL-1*β*, thus both RELA motifs appeared necessary for IL-1*β*-induced R7 transcriptional activity.

Turning to the R8 reporter, this revealed modest 3.1 ± 0.4-fold dexamethasone-dependent inducibility (*P* ≤ .001), no effect of IL-1*β* alone, but a marked 17.0 ± 3.3-fold combinatorial effect of IL-1*β*-plus-dexamethasone. Deletion of the R8 GRE motif had no effect on basal reporter activity, but prevented (*P* ≤ .001) the modest drive produced by dexamethasone alone (Fig. 4E; Supplemental Fig. 4E). Indeed, this reporter now showed 0.8 ± 0.1-fold effect of dexamethasone, that is, a modest and significant (*P* ≤ .05) repression. Similarly, the level of the response to IL-1*β*-plus-dexamethasone was markedly (*P* ≤ .001) reduced, albeit with the fold inducibility reduced to a still significant 2.4 ± 0.8-fold (*P* ≤ .01) (Fig. 4E; Supplemental Fig. 4E). Deletion of the R8 RELA motif resulted in a 3–4-fold loss (*P* ≤ .01) of basal reporter activity and completely abrogated the combinatorial response to IL-1*β*-plus-dexamethasone (Fig. 4E; Supplemental Fig. 4E). However, although the absolute level of reporter activity in response to dexamethasone was reduced compared with the wild-type reporter (Fig. 4E), owing to the reduced baseline, overall inducibility by dexamethasone was marginally enhanced (Supplemental Fig. 4E). Thus, the R8 GRE is necessary for glucocorticoid-induced reporter drive and plays a major role in the synergy with IL-1*β* with the combination treatment. Similarly, the R8 RELA motif was essential for the response to combination treatment but was not required for glucocorticoid responsiveness.

### 3.8 Effect of the dominant NF-κB inhibitor, IκBαΔN, on reporter activity

**Fig. 5:**
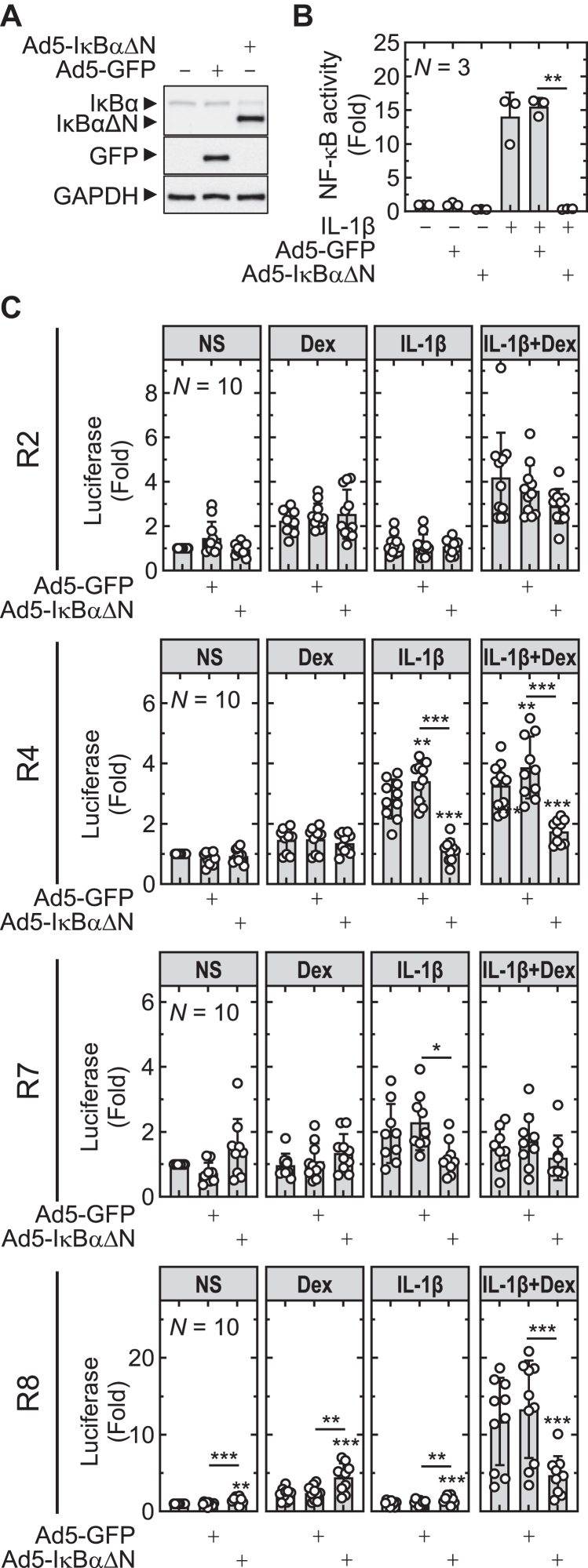
Effect of NF-*κ*B inhibition on transcriptional activity of the R2, R4, R7, and R8 reporters. (A) A549 NF-*κ*B reporter (6*κ*Btk.luc.neo) cells were either not infected or infected with Ad5-GFP, or Ad5-I*κ*B*α*ΔN at MOI 30 prior to Western blot analysis for I*κ*B*α*/I*κ*B*α*ΔN, GFP, and glyceraldehyde-3-phosphate dehydrogenase (GAPDH). (B) Naïve or virally transduced (MOI 30) NF-*κ*B reporter cells were either not stimulated or stimulated with IL-1*β* (1 ng/mL) and harvested after 6 hours for luciferase assay. (C) Naïve or virally transduced (MOI 30) R2, R4, R7, and R8 reporter constructs were either not stimulated or stimulated with dexamethasone (Dex; 1 *μ*M), IL-1*β* (1 ng/mL), or both. Cells were harvested 6 hours after treatments for luciferase assay. Data from *N* experiments were expressed as fold of not stimulated and are plotted as means ± SD overlaid with scatter plots. Significance was tested using one-way ANOVA with a Tukey’s post hoc test. ∗*P* ≤ .05, ∗∗*P* ≤ .01, ∗∗∗*P* ≤ .001 indicates significance to no stimulation or as indicated.

To examine the effect of inhibiting NF-*κ*B on the different reporters, an adenoviral construct was used to overexpress I*κ*B*α*ΔN, a dominant inhibitor of NF-*κ*B.^32^ This transduces ≥90% of A549 cells and I*κ*B*α*ΔN overexpression prevents NF-*κ*B-dependent transcription,^32^^,^^34^ as well as IL-1*β*-induced expression of both BIRC3 and BIRC2.^9^ At MOI 30, Ad5-I*κ*B*α*ΔN and Ad5-GFP infection produced I*κ*B*α*ΔN or GFP protein, respectively, and, whereas Ad5-I*κ*B*α*ΔN inhibited NF-*κ*B reporter activity induced by IL-1*β*, Ad5-GFP was without effect (Fig. 5, A and B).

Infection of R2 reporter cells with either Ad5-GFP or Ad5-I*κ*B*α*ΔN had no effect on reporter activity from unstimulated or IL-1*β*-treated cells (which alone did not affect this reporter) (Fig. 5C). Likewise, the 2.3 ± 0.5-fold induction of R2 reporter activity by dexamethasone was unaffected by either virus. However, IL-1*β* previously produced a modest *supra*-additive increase in R2 reporter activity induced by dexamethasone (Fig. 3). Thus, in naïve cells, IL-1*β* enhanced R2 reporter drive to 4.2 ± 2.0-fold in the presence of dexamethasone and this was unaffected by Ad5-GFP (Fig. 5C). With Ad5-I*κ*B*α*ΔN, IL-1*β*-plus-dexamethasone-induced reporter activity was on average decreased to 2.9 ± 0.8-fold. However, variability in this relatively weak effect did not allow significance to be reached and a clear role for NF-*κ*B was not therefore confirmed.

The R4 reporter revealed no effect of either GFP- or I*κ*B*α*ΔN-expressing virus on basal or the 1.5 ± 0.4-fold dexamethasone-induced reporter activity (Fig. 5C). However, the 2.8 ± 0.7 and 3.3 ± 0.8-fold R4 reporter activity induced by IL-1*β* or IL-1*β*-plus-dexamethasone, respectively, was unaffected by Ad5-GFP, but largely prevented (*P* ≤ .01) by Ad5-I*κ*B*α*ΔN. This supports an NF-*κ*B-dependent mechanism to induce R4 transcriptional activity by IL-1*β*. Similarly, the R7 reporter revealed 2.0 ± 0.9-fold inducibility by IL-1*β* that was prevented by Ad5-I*κ*B*α*ΔN, but not affected by Ad5-GFP (Fig. 5C). Thus, a role for NF-*κ*B in IL-1*β*-induced R7 reporter activity is supported.

As shown in Fig. 3, the R8 reporter revealed modest inducibility by dexamethasone, no effect of IL-1*β* alone, but a marked combinatorial effect with IL-1*β*-plus-dexamethasone (Fig. 3). Although the GFP expressing virus had no effect on any of these responses, Ad5-I*κ*B*α*ΔN revealed differential effects depending on the context (Fig. 5C). In unstimulated and IL-1*β*-treated cells, where IL-1*β* alone had no effect, the presence of I*κ*B*α*ΔN significantly enhanced reporter activity by ∼1.5-fold. Likewise, the modest 2.2 ± 0.8-fold inducibility by dexamethasone alone was enhanced to 4. 5 ± 1.8 by Ad5-I*κ*B*α*ΔN. However, the strong 11.7 ± 5.7-fold induction of R8 reporter activity by IL-1*β*-plus-dexamethasone combination was profoundly (*P* ≤ .001) repressed by I*κ*B*α*ΔN and is therefore consistent with a role for NF-*κ*B. Thus, although a conventional stimulatory role for NF-*κ*B is indicated in the activation of the R8 reporter by IL-1*β*-plus-dexamethasone, in absence of stimulation, or with either dexamethasone or IL-1*β* alone, an inhibitory role is suggested.

### 3.9 Effect of small molecule IκB kinase inhibitors on reporter activation

**Fig. 6:**
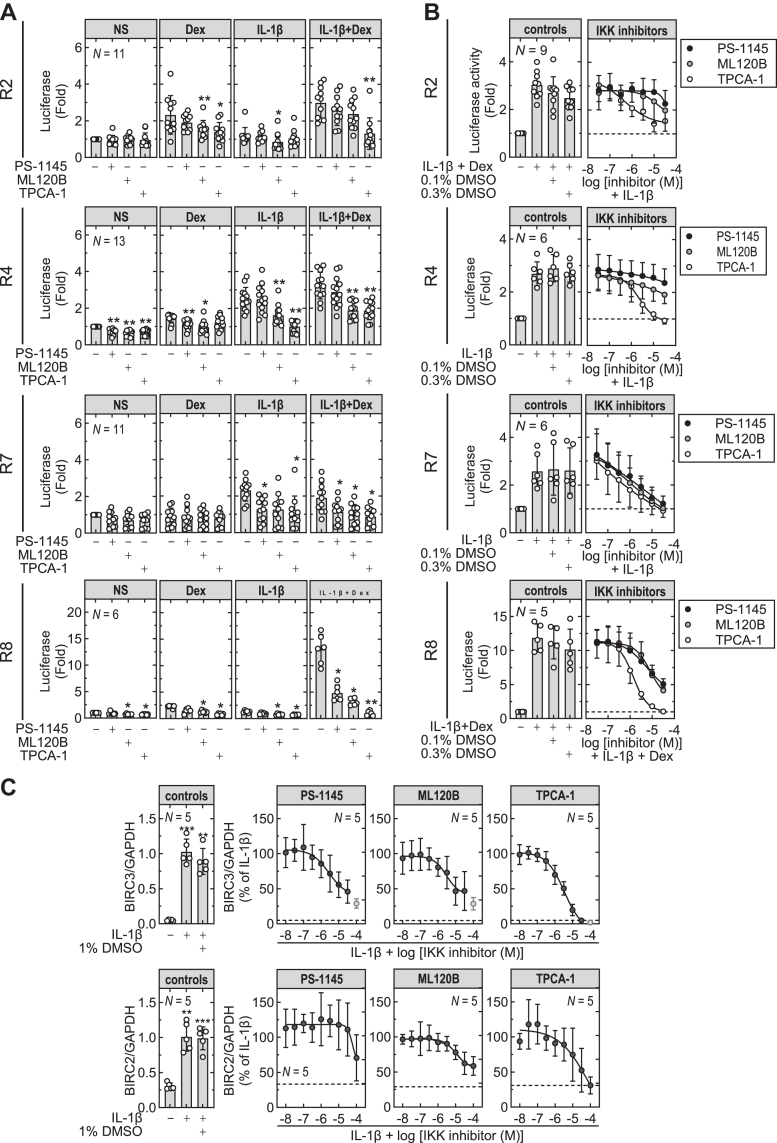
Effect of IKK*β* inhibitors on R2, R4, R7, and R8 reporter activity and BIRC3/BIRC2 mRNA expression. (A) R2, R4, R7, and R8 reporter cells were pretreated, or not, with PS-1145 (30 *μ*M), ML-120B (30 *μ*M), or TPCA-1 (30 *μ*M) for 90 minutes prior to treatment, or not, with dexamethasone (Dex; 1 *μ*M), IL-1*β* (1 ng/mL), or both. Cells were harvested 6 hours after treatments for luciferase assay. (B) R2, R4, R7, and R8 reporter cells were pretreated, or not, with the indicated concentrations of DMSO (v/v), PS-1145, ML120B, or TPCA-1 for 90 minutes prior to no stimulation or treatment with IL-1*β* or IL-1*β* + dexamethasone, as indicated. Cells were harvested 6 hours after treatments for luciferase assay. (C) A549 cells were treated as in (B) and harvested after 2 hours for RNA extraction and qPCR analysis of BIRC3, BIRC2, and glyceraldehyde-3-phosphate dehydrogenase (GAPDH) mRNAs. Luciferase activity data were expressed as fold of not stimulated. BIRC3 and BIRC2 mRNA data were normalized to GAPDH and plotted as gene/GAPDH or, for curve fitting, as a percentage of IL-1*β*-treated. Data from *N* experiments were plotted as mean ± SD overlaid with scatter plots or with fitted curves. Significance was tested using one-way ANOVA with a Tukey’s post hoc test. ∗*P* ≤ .05, ∗∗*P* ≤ .01, ∗∗∗*P* ≤ .001 indicates significance to unstimulated or as indicated.

To explore possible roles for I*κ*B kinases (IKKs), effects of IKK*β*-selective inhibitors, PS-1145, ML120B, and TPCA-1,^50^, ^51^, ^52^, ^53^, ^54^ were tested on the R2, R4, R7, and R8 reporters. These compounds inhibit IL-1*β*-induced NF-*κ*B reporter activity with EC_50_ values of 1–2, 8.2, and 2.0 *μ*M, respectively.^55^, ^56^, ^57^ As each compound produces near maximal effects at 30 *μ*M, this concentration was adopted for initial investigations (Fig. 6A).

The R2 reporter revealed no effect of PS-1145 (30 *μ*M) on basal reporter activity or in the presence of IL-1*β*, which alone had no effect (Fig. 6A). Similarly, dexamethasone- or dexamethasone-plus-IL-1*β*-induced reporter activity was unaffected by PS-1145. ML120B (30 *μ*M) produced modest, 34.0% (*P* ≤ .01) and 27.8% (*P* ≤ .05), reductions in reporter activity induced by dexamethasone or in the presence of IL-1*β*. However, the lack of effect for PS-1145, which is effective at IKK*β* inhibition,^50^, ^51^, ^52^, ^53^, ^54^ suggests off-target effects of ML120B. Indeed, when activated by IL-1*β*-plus-dexamethasone, neither PS-1145 nor ML120B affected R2 reporter activity until at least 10 *μ*M (Fig. 6B), which further supports the concept of off-target effects. Similarly, as IKK*β* and NF-*κ*B are not activated by dexamethasone, the inhibition by TPCA-1 (30 *μ*M) of dexamethasone-induced reporter activity is likely to also represent an off-target effect. Further, the lack of effect of PS-1145 and ML120B on R2 reporter activity induced by IL-1*β*-plus-dexamethasone suggests a major role for IKK*β* is unlikely (Fig. 6B; Table 2). Nevertheless, the effect (*P* ≤ .01) of TPCA-1, with 56% inhibition, was more profound and dosing of this compound revealed a relatively high potency (pEC_50_ 6.35 ± 0.35) (Fig. 6B; Table 2). Because TPCA-1 is only weakly IKK*β*-selective relative to IKK*α*, these data raise the possibility of a role for IKK*α*.^57^^,^^58^

**Table 2:** Potency of IKK inhibitors on activity of the R2, R4, R7, and R8 *BIRC3*/*BIRC2* reporters and BIRC2 and BRIC3 mRNA expression induced by IL-1*β* Data from the concentration responses analyses in Fig. 6 were subjected to 4-parameter curve fitting using GraphPad Prism v11 to produce overall pEC_50_ values, each with an associated SE.

Reporter/Gene mRNA	IKK Inhibitor					
	PS-1145		ML120B		TPCA-1	
	pEC_50_ (−log[M] ± SE)	*N*	pEC_50_ (−log[M] ± SE)	*N*	pEC_50_ (−log[M] ± SE)	*N*
R2	Wide^a^	9	4.84 ± 1.41	9	6.35 ± 0.35	9
R4	Wide^a^	6	Wide^a^	6	5.65 ± 0.19	6
R7	Wide^a^	6	5.93 ± 0.80	6	Wide^a^	6
R8	5.24 ± 0.53	5	5.00 ± 0.19	5	5.86 ± 0.11	5.
BIRC2	wide	5	4.98 ± 0.27	5	Wide^a^	5
BIRC3	5.56 ± 0.44	5	5.53 ± 0.32	5	5.86 ± 0.15	5

a pEC_50_ was either highly variable (SE ≥2) or could not be determined.

Basal R4 reporter activity was reduced 38.7%–27.6% (all *P* ≤ .01) by each IKK inhibitor (30 *μ*M), whereas the low level of dexamethasone-induced reporter activity was reduced 24.5% and 34.4% by PS-1145 and ML120B (*P* ≤ .01 and .05), respectively, but not by TPCA-1. Further, a lack of IKK activation either in untreated cells or in the presence of dexamethasone suggests that roles for IKK inhibition are unlikely and again is indicative of off-target effects. With IL-1*β* and IL-1*β*-plus-dexamethasone-induced R4 activity, both ML120B and TPCA-1 significantly (all *P* ≤ .01) inhibited reporter activity. With TPCA-1, this produced a pEC_50_ of 5.65 (EC_50_ 2.6 *μ*M) and resulted in near complete ablation of IL-1*β*-induced reporter activity (Fig. 6B). By contrast, the effect of ML120B was lesser and required considerably higher (≥30 *μ*M) concentrations to reach EC_50_ levels of inhibition (Fig. 6B). This, combined with the lack of effect for PS-1145, suggests that IKK*β* inhibition does not affect IL-1*β*-induced R4 reporter activity. However, the inhibition by TPCA-1 is again consistent with additional IKK*β*-independent effects that could involve IKK*α*.^57^^,^^58^

The R7 reporter showed 2.3-fold inducibility by IL-1*β* that was attenuated slightly in the additional presence of dexamethasone. The 3 IKK inhibitors (30 *μ*M) revealed no effect on either untreated or dexamethasone-stimulated cells (Fig. 6A). However, in the context of IL-1*β* all 3 compounds reduced IL-1*β*-induced reporter activity by 45%–51.7% (*P* ≤ .05 for PS-1145 and TPCA-1), that is, to near basal levels. Similarly, with IL-1*β*-plus-dexamethasone, R7 reporter activity was reduced (all *P* ≤ .05) by the 3 inhibitors. This supports a role for IKK*β* in activation of the R7 reporter by IL-1*β*. However, analyses of inhibitor concentration on IL-1*β*-induced R7 reporter activity revealed remarkably linear concentration-response relationships for all 3 compounds (Fig. 6B). Thus, although not discounting a role for IKK*β*, the data clearly indicate effects at multiple pharmacological targets.

With the R8 reporter, PS-1145 (30 *μ*M) revealed no effect on untreated or IL-1*β*- or dexamethasone-treated cells (Fig. 6A). However, in R8 reporter cells treated with IL-1*β*-plus-dexamethasone, PS-1145 produced >60% inhibition (*P* ≤ .05) of reporter drive. Similarly, ML120B (30 *μ*M) inhibited IL-1*β*-plus-dexamethasone-induced R8 reporter activity by >75% (*P* ≤ .05), whereas complete inhibition (*P* ≤ .01) was observed with TPCA-1 (30 *μ*M). However, both ML120B and TPCA-1 also resulted in modest, but significant (all *P* ≤ .05) loss of basal, dexamethasone- and IL-1*β*-stimulated reporter activity. Given the lack of effect of PS-1145 on basal and both IL-1*β*- and dexamethasone-treated cells, it is likely that these effects of ML120B and TPCA-1 are not due to IKK*β* inhibition. Conversely, the concentration-dependent effects of PS-1145, ML120B, and TPCA-1 on the response to IL-1*β*-plus-dexamethasone are consistent with inhibition of NF-*κ*B and therefore support a role for IKK*β* (Fig. 6B). The enhanced effectiveness of TPCA-1 is again indicative of an additional pharmacological target, potentially, IKK*α*.^57^^,^^58^

### 3.10 Effect of IKK inhibitors on BIRC3 and BIRC2 mRNA expression

A549 cells were stimulated with IL-1*β* for 2 hours, a time at which BIRC3 and BIRC2 mRNAs were highly induced by IL-1*β*, but before the peak of mRNA expression.^9^ As expected, IL-1*β* induced expression of both BIRC3 and BIRC2 mRNAs (*P* ≤ .001 and .01, respectively) and these responses were unaffected by DMSO at 1% (Fig. 6C). Both PS-1145 and ML120B produced concentration-dependent (pEC_50_ 5.56 and 5.53, respectively), but partial (∼54% repression at 30 *μ*M) effects on IL-1*β*-induced BIRC3 mRNA (Fig. 6C; Table 2). Given close similarity with the inhibition of NF-*κ*B-dependent transcription induced by IL-1*β*,^55^^,^^56^ these data are consistent with effects due to IKK*β* inhibition. TPCA-1 resulted in a near complete inhibition of IL-1*β*-induced BIRC3 mRNA that occurred with a pEC_50_ of 5.47 (Fig. 6C; Table 2). Although similar effects were also apparent on an NF-*κ*B reporter,^56^ the additional efficacy of TPCA-1 is again consistent with effects at an additional pharmacological target such as IKK*α*.^57^^,^^58^ With BIRC2 mRNA, PS-1145 showed no effect on IL-1*β*-induced expression until a concentration of 100 *μ*M (Fig. 6C; Table 2). ML120B resulted in a partial repressive effect with a pEC_50_ of 4.98, whereas below 10 *μ*M, TPCA-1 was without effect (Fig. 6C; Table 2). These data do not therefore support roles for IKK*β* in the ability of IL-1*β* to induce BIRC2 mRNA.

### 3.11 Antagonism by Org34517 supports a role for GR

**Fig. 7:**
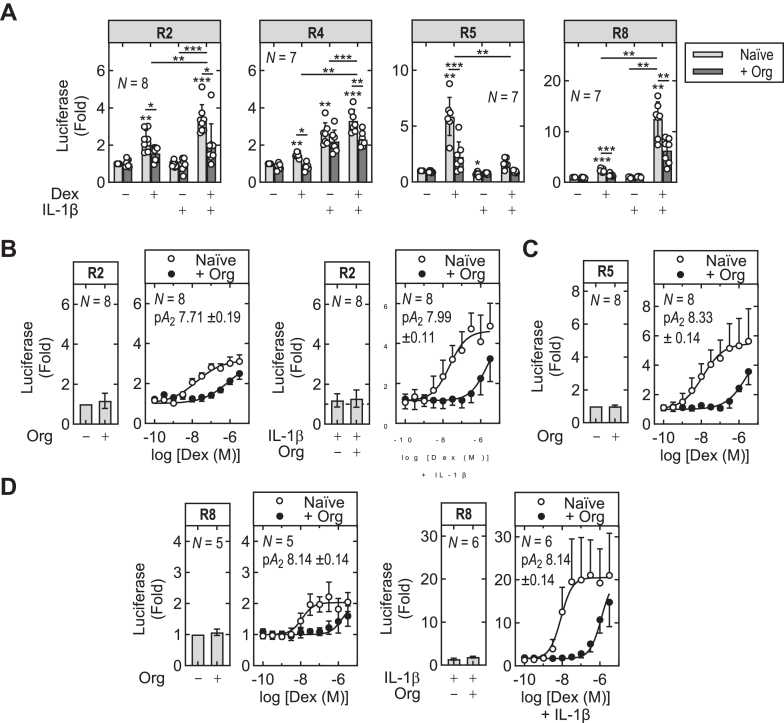
Antagonism of dexamethasone-induced reporter activity by Org34517. (A) The R2, R4, R5, and R8 reporter constructs were incubated with Org34517 (Org; 1 *μ*M) for 60–90 minutes prior to treatment with dexamethasone (Dex; 1 *μ*M), IL-1*β* (1 ng/mL), or both. (B–D) The R2, R5, and R8 reporters were pretreated, or not, with Org34517 (1 *μ*M) prior to stimulation with the indicated concentrations of dexamethasone, without or with IL-1*β*, as indicated. Cells were harvested 6 hours after treatments for luciferase assay. Data from *N* experiments were expressed as fold of not stimulated and are plotted as means ± SD overlaid with scatter plots or with fitted curves. Significance was tested using one-way ANOVA with a Tukey’s post hoc test. ∗*P* ≤ .05, ∗∗*P* ≤ .01, ∗∗∗*P* ≤ .001 indicates significance to not stimulated or otherwise indicated.

In initial experiments, the R2, R4, R5, and R8 reporters were stimulated with IL-1*β* (1 ng/mL) or/and dexamethasone (1 *μ*M), each in the absence or presence of the competitive GR antagonist, Org34517 (1 *μ*M).^43^^,^^59^ The R2 reporter revealed activation by dexamethasone with a greater effect of IL-1*β*-plus-dexamethasone, responses that were both reduced (*P* ≤ .05) by Org34517 (Fig. 7A). There was no effect of Org34517 alone or in the presence of IL-1*β*, which was also without effect. At the R4 reporter, dexamethasone, IL-1*β* and IL-1*β*-plus-dexamethasone each induced reporter activity and these responses were reduced (*P* ≤ .05 and .01, respectively) by Org34517 (Fig. 7A). With the R5 reporter, dexamethasone increased luciferase activity 5.9-fold (*P* ≤ .01) and this was reduced to 1.7-fold (*P* ≤ .001) by Org34517. Basal, IL-1*β*- and IL-1*β*-plus-dexamethasone treatments were not significantly affected by Org34517. The 2.5-fold (*P* ≤ .001) inducibility by dexamethasone of the R8 reporter was largely prevented (to 1.4-fold, *P* ≤ .001) by Org34517 and the 12.5-fold induction produced by IL-1*β*-plus-dexamethasone was reduced to 6.3-fold (*P* ≤ .01) by Org34517.

Although the above data support roles for GR in the effects of dexamethasone at the R2, R4, R5, and R8 reporters, Schild analysis was also performed using Org34517 on the R2, R5, and R8 reporters. The very modest effect of dexamethasone on R4 was deemed ill-suited for such analysis. Analysis of the R2 construct revealed concentration-dependent increases by dexamethasone that were enhanced by IL-1*β*, with IL-1*β* alone having no effect (Fig. 7B). The responses to dexamethasone and dexamethasone-plus-IL-1*β* revealed surmountable antagonism by Org34517. In each case, Schild analysis produced pA_2_ values of 7.71 and 7.99 that are consistent with antagonism at GR.^43^ With the R5 reporter, dexamethasone-induced reporter activity (pEC_50_ 7.93 ± 0.26) was abrogated by Org34517 for all dexamethasone concentrations below 0.1 *μ*M (Fig. 7C). Above this concentration, dexamethasone progressively overcame the effect of Org34517, giving a pA_2_ of 8.33 ±0.14. Although this is consistent with antagonism at GR,^43^ full reversal of the Org34517 effect was not achieved. With the R8 reporter, the modest ∼2-fold induction by dexamethasone (pEC_50_ 7.97 ± 0.09) was competitively antagonized by Org34517 to give a pA_2_ of 8.14 ± 0.11 (Fig. 7D). Similarly, the enhanced response in the presence of IL-1*β* produced a pEC_50_ of 8.08 ± 0.13 for dexamethasone and showed competitive antagonism with Org34517 to give a pA_2_ of 8.14 ± 0.11. Thus, these Schild analyses provide enhanced support for competitive antagonism by Org34517 acting at GR in respect of the R2, R5, and R8 reporters.

### 3.12 Dexamethasone-induces chromatin rearrangement at the *BIRC3*/*BIRC2* locus

**Fig. 8:**
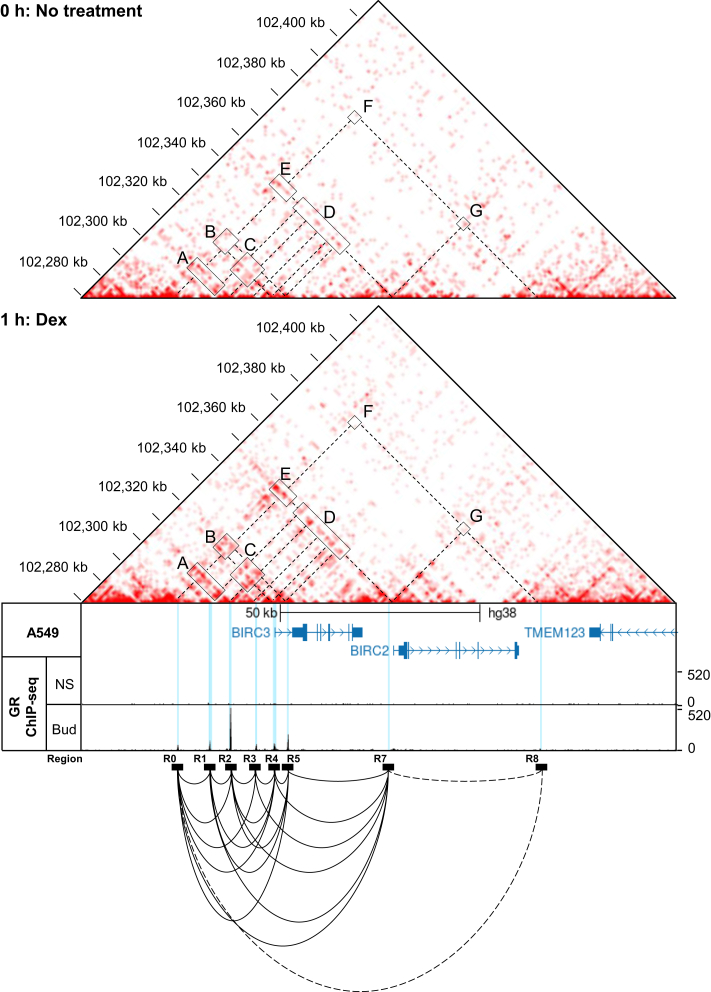
Interactions within the *BIRC3*/*BIRC2* locus in the absence and presence of glucocorticoid. Hi-C analysis of A549 cells treated with or without dexamethasone (Dex; 100 nM) for 1 hour is displayed as interaction matrices (pyramids) from publicly accessible data.^41^^,^^60^ This corresponds to ∼150 kb (chr11:102,269,255-102,418,532) spanning the regions R0–R8 within the *BIRC3*/*BIRC2* locus. Images were generated using the JuiceBox tool from the Genomics of Gene Regulation project.^41^ The intensity of the color reflects the number of ligation events and is indicative of the interaction frequency between any 2 loci joined by downward 45° sloping lines. Interaction hotspots (A–G) within the *BIRC3*/*BIRC2* region are annotated with dashed lines. Below depicts a genome browser snapshot of GR ChIP-seq traces in A549 cells following budesonide (Bud; 300 nM) treatment for 1 hour that spans the same ∼150 kb of the *BIRC3*/*BIRC2* locus. Regions R0–R8 are highlighted in with tentative interaction loops in solid black lines (below). Dotted lines highlight absence of interactions involving R8.

Publicly accessible chromosome conformation capture data, available via the Genomics of Gene Regulation project and derived from Hi-C data experiments in A549 cells treated with dexamethasone,^41^^,^^60^ were interrogated at the *BIRC3*/*BIRC2* locus. Chromatin interaction matrices for cells that were untreated and treated with dexamethasone for 1 hour were visualized using the “Juicebox” webtool,^42^ such that color intensity reflects the frequency of ligation events in the chromosome conformation capture experiment and indicates the interaction frequency between the genomic loci linked by downward 45° sloping lines from any point (Fig. 8). Sites of potential interaction in respect of the *BIRC3*/*BIRC2* GBR/RBRs were annotated as A–G. In the absence of dexamethasone, there were relatively low levels of interaction indicated for any site. However, the presence of weak signals nevertheless suggests basal interactions that occurred between the broader R0 region, that is, R0 plus other more 5′ sequences, with R1 plus R2; R2 with R4 and R5; R0, R4, and R5 with R7; and weakly between R7 and R8. With the exception of R8, these data correlate well with the H1-hESC and HFFc6 cell micro-C data and collectively suggest genuine interaction between these regions and the TSSs for both genes (Fig. 1A). In the presence of dexamethasone, increased signal intensity was observed for multiple sites (Fig. 8). Intensity enhancements at the A, B, C, D, and E sites indicate increased associations between the regions R0–R5, as well as with R7. Thus, increased intensity at site A shows the broader R0 region interacting with R1 and R2. Color increases at site B suggest interactions with the broader R0 region and R4 and R5, whereas site C indicates interactions between R1 plus R2 with R4 and R5. Elevated signal at sites D and E further suggest that not only do R1–R5 interact with R7, but that the broader R0 is also recruited. Given the locations of R4 and R7, proximal to the *BIRC3* and *BIRC2* TSSs, respectively, these data support the assembly of 3D structures involving transcriptionally active RBRs and GBRs in a manner that are positioned to promote *BIRC3* and *BIRC2* gene transcription. Notably, there was no increased intensity at either F or G suggesting no interaction between these regions and R8 (Fig. 8).

### 3.13 Coregulation of apoptotic/antiapoptotic genes by IL-1β and glucocorticoid

**Fig. 9:**
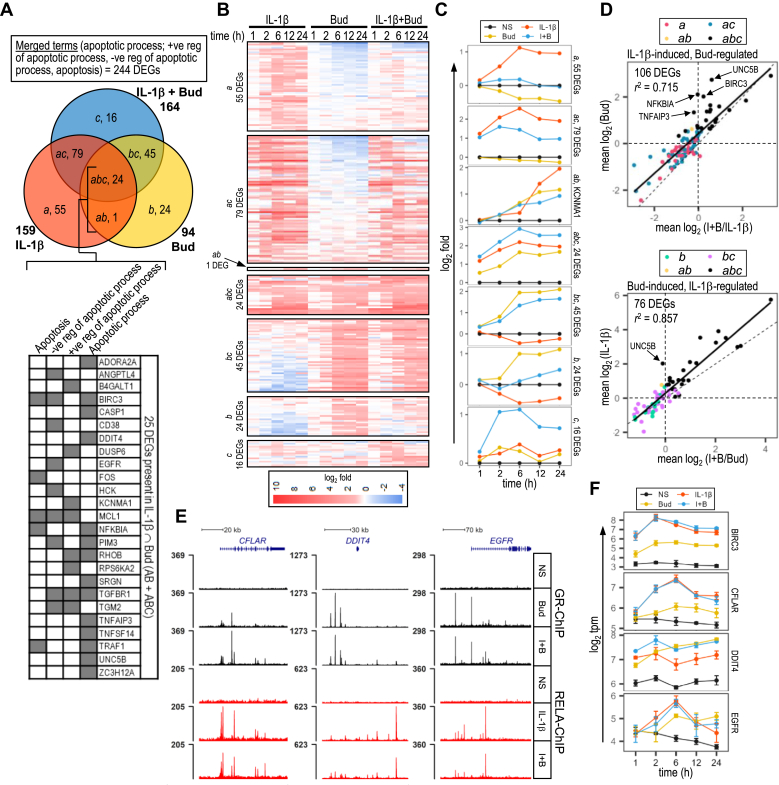
Regulation of DEGs implicated in the control of apoptotic processes by IL-1*β* and budesonide. (A) DEGs that were upregulated (≥2-fold, *q* ≤ 0.05) by IL-1*β* (1 ng/mL), budesonide (Bud; 300 nM), or the combination of both (IL-1*β* + Bud) at any time (1, 2, 6, 12, or 24 hours) and were associated with the GO terms “apoptotic process,” “positive (+ve) regulation (reg) of apoptotic process,” “negative (−ve) regulation of apoptotic process,” or the KEGG pathway term “Apoptosis” were plotted as a Venn diagram. Total DEG number is indicated for each treatment along with DEG numbers within each overlap/region (*a*, *b*, *c*, *ab*, *ac*, *bc,* and *abc*). The DEGs present in the IL-1*β* ∩ budesonide intersection (*abc* + *ab*) is shown for each of the 4 GO terms. (B) Average (*N* = 4) log_2_ fold values for each DEG were plotted as heat maps for all DEGs within each region of the Venn diagram in (A). DEGs in *a* and *ac* were ranked (low-to-high) according to the average effect for all times of budesonide alone and DEGs in *b* and *bc* were ranked (high-to-low) according to the average effect for all times of IL-1*β* alone. DEGs in *abc* and *c* were ordered by hierarchical clustering. (C) Expression (as log_2_ fold) for all DEGs within each group was summarized for each treatment (NS = not stimulated, IL-1*β*, Bud = budesonide, I + B = IL-1*β* + budesonide) as a mean and plotted for each time. (D) Relationship between the change in response on combination treatment relative to each monotreatment was examined as a function of the other monotreatment. (Upper panel) Mean log_2_ fold for IL-1*β* + budesonide/fold for IL-1*β* for all time points was plotted against the mean log_2_ fold for all time points in respect of the 106 IL-1*β* upregulated (≥2-fold, *q* ≤ 0.05) DEGs that were independently regulated by budesonide (Bud; *q* ≤ 0.05). (Lower panel) Mean log_2_ fold for IL-1*β* + budesonide/fold for IL-1*β* for all time points was plotted against the mean log_2_ fold for all time points in respect of the 76 budesonide-upregulated (≥2-fold, *q* ≤ 0.05) DEGs that were independently regulated by IL-1*β* (*q* ≤ 0.05). (E) Normalized ChIP-seq data showing GR (black) and RELA (red) recruitment for the indicated loci in A549 cells that were not stimulated (NS) or IL-1*β*, budesonide (Bud) or IL-1*β* + budesonide (I + B) treated, as described in Fig. 1A. (F) mRNA expression data (*N* = 4) are plotted as log_2_ transcripts/million (tpm) for the indicated DEGs to show transcript levels following no stimulation (NS) or treatment with IL-1*β*, budesonide (Bud) or IL-1*β* + budesonide (I + B), as in panel (A).

To more generally explore the ability of inflammatory cytokines and glucocorticoids to upregulate expression of genes involved in apoptosis, mRNA sequencing data from A549 cells treated with IL-1*β* (1 ng/mL) or/and budesonide (300 nM) for 1, 2, 6, 12, and 24 hours was used to identify DEGs showing ≥2-fold upregulation (*q* ≤ 0.05) by IL-1*β*, budesonide and IL-1B-plus-budesonide. Depending on time point, some DEGs showed both upregulation and downregulation but were retained in the current analysis. Following GO enrichment analysis for each IL-1*β*-, budesonide-, and IL-1*β*-plus-budesonide-induced gene list, GO terms containing “apoptosis” or “apoptotic” were extracted (Supplemental Table 2). This revealed 10, 2, and 7 GO terms, respectively, with Benjamini significance (*P*_*B*_ ≤ .05) and the 3 biological process terms (“*apoptotic process,*” “*positive regulation of apoptotic process,*” and “*negative regulation of apoptotic process*”) containing the greatest DEG counts for each treatment, as well as the KEGG pathway term “*Apoptosis*” were selected for further analysis (Supplemental Table 2). Within each treatment ∼70% of DEGs were unique to each term and merging the 3 treatment groups revealed that overall 72.1% of DEGs were specific to a single GO term (Supplemental Table 3; Supplemental Fig. 5). Therefore, to achieve greatest representation, the DEGs from all 4 GO terms were combined to give 244 DEGs where 25 were common between IL-1*β*- and budesonide-upregulated DEGs (Fig. 9A). At 15.7% and 26.6% of the IL-1*β*- and budesonide-induced DEGs, respectively, χ^2^-testing shows this overlap to be vastly more than expected by simple chance (Supplemental Fig. 6A). Equivalent overlaps were also apparent for each individual GO term and together this implies biological relevance for co-upregulation (Supplemental Fig. 6B). Further, with the 25 DEGs in this IL-1*β*/budesonide intersection, GO enrichment showed the regulation of NF-*κ*B, a key regulator of (anti-) apoptotic effects,^61^^,^^62^ to be prominent (Fig. 9A; Table 3). Thus, DEGs for *NFKBIA*, or I*κ*B*α*, *TNFAIP3*, or A20, the adaptor, *TRAF1*, the caspase, *CASP1*, or the TNF receptor ligand, *TNFSF14*, in addition to *BIRC3* all modulate NF-*κ*B activity and are all independently upregulated by both IL-1*β* and budesonide. The presence of kinases (*HCK* and *PIM3*), growth factor receptors (*TGFBR1* and *EGFR*), other glycoproteins (*ANGPLL4* and *CD38*), the BCL2 family member, *MCL1*, or the DNA damage-inducible transcript, *DDIT4*, further illustrates the potential for co-upregulated gene expression to influence apoptotic processes.

**Table 3:** GO enrichment analysis of the 25 DEGs in the IL-1*β*-budesonide intersection (IL-1*β* ∩ bud) The 25 DEGs in the IL-1*β*-budesonide intersection (IL-1*β* ∩ bud) were submitted into the Database for Annotation, Visualization, and Integrated Discovery. The top 10 GO terms relating to biological process or KEGG pathway were ranked by Benjamini significance (*P*_*B*_). Fold enrichment (Fold) and DEG count (Count) is shown.

ID	Term	Count	Fold	*P*_*B*_
GO:0006915	Apoptotic process	14	17.5	3.2 × 10^−11^
GO:0043065	Positive regulation of apoptotic process	8	19.0	1.9 × 10^−5^
GO:0043066	Negative regulation of apoptotic process	9	13.8	1.9 × 10^−5^
hsa04064	NF-*κ*B signaling pathway	5	19.2	.011
hsa04668	TNF signaling pathway	5	16.9	.011
hsa04210	Apoptosis	5	14.8	.012
GO:0007165	Signal transduction	8	4.7	.12
GO:0030154	Cell differentiation	6	6.8	.12
GO:0001666	Response to hypoxia	4	17.5	.12
GO:0042311	Vasodilation	3	49.6	.12

To explore the ability of IL-1*β* and budesonide to coregulate expression of the above 244 DEGs, heat maps were generated for each region of the Venn diagram in Fig. 9A (Fig. 9B; Supplemental Fig. 7). In region *a*, the 55 DEGs upregulated by IL-1*β* were, as illustrated by the summary plot (Fig. 9C), strongly repressed following budesonide cotreatment. The 79 IL-1*β* and IL-1*β*-plus-budesonide-upregulated, but not budesonide-regulated, DEG group, *ac*, revealed a generally greater fold induction by IL-1*β* that was less attenuated on cotreatment (Fig. 9C). GO associated with region *a* revealed numerous kinases, regulation of signaling and transcription as relates to both inflammation and cell fate/apoptosis (Table 4). Likewise, the DEGs in *ac* showed enrichment of signaling and transcription, especially as related to inflammation and the NF-*κ*B pathway (Table 4). The 25 DEGs in the IL-1*β*/budesonide-upregulated overlap (regions *abc* and *ab)* were, with the exception of the potassium calcium-activated channel, a maxi K channel, *KCNMA1*, all within region *abc* and therefore showed inducibility by IL-1*β*-plus-budesonide. Indeed, the effect of IL-1*β*-plus-budesonide was on average greater than for each treatment alone (Fig. 9, B and C). Again, GO associated with these coregulated DEGs was heavily focused on the NF-*κ*B pathway, inflammation and related signaling (Table 4). Turning to DEGs showing more prominent upregulation by budesonide, region *bc* revealed strong budesonide-upregulation that was on average modestly reduced by cotreatment (Fig. 9, B and C). Likewise, DEGs in region *b* that were only upregulated upon budesonide treatment were less highly induced by budesonide and were much more reduced on cotreatment. GO enriched with DEGs in both regions *bc* and *b* revealed regulation (negative and positive) of transcription and signaling to transcription in addition to terms for proliferation, cancer and apoptosis/cell death (Table 4). Finally, in region *c*, each of IL-1*β* and budesonide produced modest increases in gene expression that when combined resulted in elevated expression such that expression of these 16 DEGs now satisfied the inclusion threshold for expression (Fig. 9, B and C). GO enrichment for this region revealed terms for the regulation of transcription, signaling, plus cell maturation, proliferation and apoptosis (Table 4).

**Table 4:** GO enrichment showing the top 5 GO terms associated with the DEGs in each region of the IL-1*β*, budesonide, and IL-1*β*-plus-budesonide-upregulated DEG Venn diagram DEG lists for each region of the Venn diagram depicting overlap in the upregulated DEGs consequent on IL-1*β*, budesonide, or IL-1*β*-plus-budesonide treatment were submitted to the Database for Annotation, Visualization, and Integrated Discovery and the top 5 most enriched terms for biological process or KEGG pathway are shown. GO terms for “apoptotic process,” “positive regulation of apoptotic process,” “negative regulation of apoptotic process,” and the KEGG pathway term “Apoptosis” were excluded being entry criteria for all these DEGs. DEG count (Count), fold enrichment (Fold), and Benjamini significance (*P*_*B*_) are shown.

Region	ID	Term	Count	Fold	*P*_*B*_
*a*	GO:0006468	Protein phosphorylation	9	10.9	2.7 × 10^−4^
	hsa05417	Lipid and atherosclerosis	9	8.2	8.3 × 10^−4^
	GO:0010628	Positive regulation of gene expression	10	7.0	1.5 × 10^−3^
	hsa05221	Acute myeloid leukemia	6	17.4	9.1 × 10^−4^
	hsa05164	Influenza A	8	9.1	9.1 × 10^−4^
*ac*	hsa04668	TNF signaling pathway	14	16.8	1.8 × 10^−10^
	GO:0045944	Positive regulation of transcription by RNAP2	27	5.3	3.9 × 10^−10^
	hsa05164	Influenza A	15	12.4	4.6 × 10^−10^
	GO:0006954	Inflammatory response	17	9.9	3.0 × 10^−9^
	GO:0008285	Negative regulation of cell population proliferation	17	9.6	3.7 × 10^−9^
*abc*	hsa04064	NF-*κ*B signaling pathway	5	16.8	9.0 × 10^−3^
	hsa04668	TNF signaling pathway	5	5.3	9.0 × 10−3
	GO:0007165	Signal transduction	8	12.4	8.7 × 10^−2^
	GO:0030154	Cell differentiation	6	9.9	1.2 × 10^−1^
	GO:0006954	Inflammatory response	5	9.60	1.3 × 10^−1^
*bc*	GO:0000122	Negative regulation of transcription by RNAP2	12	5.4	1.1 × 10^−3^
	GO:0032355	Response to estradiol	5	24.8	5.6 × 10^−3^
	GO:0008285	Negative regulation of cell population proliferation	8	7.9	5.6 × 10^−3^
	hsa05202	Transcriptional misregulation in cancer	7	9.5	5.0 × 10^−3^
	hsa04068	FoxO signaling pathway	6	12.1	5.0 × 10^−3^
*b*	GO:0001666	Response to hypoxia	5	22.7	7.2 × 10^−3^
	GO:0048144	Fibroblast proliferation	3	60.7	9.3 × 10^−2^
	GO:2000117	Negative regulation of cysteine-type endopeptidase activity	3	60.7	9.3 × 10^−2^
	GO:0045893	Positive regulation of DNA-templated transcription	6	6.77	1.0 × 10^−1^
	GO:0062099	Negative regulation of programmed necrotic cell death	2	809	1.6 × 10^−1^
*c*	GO:0045944	Positive regulation of transcription by RNAP2	9	8.8	2.2 × 10^−4^
	GO:0010628	Positive regulation of gene expression	7	16.8	2.2 × 10^−4^
	GO:0048469	Cell maturation	4	147	2.7 × 10^−4^
	GO:0043066	Negative regulation of apoptotic process	6	14.4	3.0 × 10^−3^
	hsa05205	Proteoglycans in cancer	5	18.1	8.9 × 10^−3^

### 3.14 Correlation between regulation by each treatment alone and effect in combination

Ranking the IL-1*β*-upregulated DEGs in regions *a* and *ac* by the effect of budesonide alone (low-to-high upregulation) revealed how budesonide may influence the IL-1*β* response on cotreatment (Fig. 9B). Many IL-1*β*-upregulated DEGs that showed independent repression by budesonide appeared to show reduced expression, relative to IL-1*β* alone, with cotreatment. Conversely, IL-1*β*-upregulated DEGs that were independently increased by budesonide showed less repression or were modestly enhanced in the combination treatment relative to IL-1*β* alone (Fig. 9B). Similarly, many budesonide-upregulated DEGs in *b* and *bc* revealed independent regulation by IL-1*β* that appeared predictive of effects in the combination treatment (Fig. 9B). To formally explore these effects, the 106 DEGs upregulated by IL-1*β* (*q* ≤ 0.05; ≥2-fold), but which also showed significant (*q* ≤ 0.05) independent upregulation or downregulation by budesonide were extracted and average log_2_(fold) for budesonide was plotted against the average log_2_(IL-1*β*-plus-budesonide/IL-1*β*) (Fig. 9D, upper panel). This revealed a striking relationship, in which an *r*^2^ of 0.715 confirms independent regulation by budesonide to be highly predictive of the effects of the combination treatment on IL-1*β*-upregulated DEGs. Performing an equivalent analysis for the 76 budesonide-upregulated (≥2-fold, *q* ≤ 0.05) DEGs that were independently upregulated or downregulated by IL-1*β* (*q* ≤ 0.05) revealed a similar correlation (*r*^2^ = 0.857) (Fig. 9D, lower panel), one that confirms a key role for independent regulation by IL-1*β* in determining the overall response to IL-1*β*-plus-budesonide.

### 3.15 Loci for coregulated DEGs reveal RELA and GR recruitment

DEGs within region *abc* of Fig. 9A were independently upregulated by both IL-1*β* and budesonide and their expression remained upregulated by IL-1*β*-plus-budesonide cotreatment. This group contains various regulators of the NF-*κ*B pathway including NFKBIA and TNFAIP3 that, along with BIRC3 in the current study, are known to recruit both RELA and GR to their gene loci.^22^^,^^63^^,^^64^ Thus, as is also evident for *DDIT4* and *EGFR* (Fig. 9, E and F), or *CD38*, *B4GALT1*, *PIM3*, and *UNC5B* (Supplemental Fig. 8), it was not surprising to find IL-1*β* and budesonide-induced recruitment of RELA and/or GR to many, if not most, of the loci for DEGs in this *abc* region. Similarly, within the IL-1*β*-induced groups (*a* and *ac*), independent upregulation by budesonide clearly occurred, albeit below the 2-fold threshold (Fig. 9B; Supplemental Fig. 7). This was apparent for genes including *CFLAR* (Fig. 9F), a major driver of antiapoptotic effects,^65^ which also recruited GR and RELA with each treatment and with IL-1*β*-plus-budesonide (Fig. 9E). Similarly, many budesonide-upregulated DEGs, in regions *bc* and *c*, were maintained in the context of IL-1*β*-plus-budesonide, plus also showed a lesser independent upregulation by IL-1*β* alone. For example, SGK1 and ERRFI1 mRNAs were strongly induced by budesonide but more modestly (<2-fold) upregulated by IL-1*β* and each locus recruited GR and RELA in response to budesonide and IL-1*β*, respectively (Supplemental Fig. 8).

Thus, as illustrated by *CD38*, *B4GALT1*, *CFLAR*, *DDIT4*, *ERRFI1*, *EGFR*, *PIM3*, *SGK1*, and others, the independent recruitment of each of GR and/or RELA to these loci is consistent with direct transcriptional upregulation by budesonide and/or IL-1*β*, respectively. However, in the context of IL-1*β*-plus-budesonide overall outcomes on gene expression ranged from markedly enhanced combinatorial regulation to, as apparent for BIRC3 mRNA, less than additivity (Fig. 9F; Supplemental Fig. 8). This outcome was evident for many of the IL-1*β*-upregulated DEGs (eg, BIRC3, NFKBIA, TNFAIP3, and UNC5B) that were also upregulated by budesonide; however, combination of IL-1*β*-plus-budesonide resulted in effects that were similar to IL-1*β* alone (ie, mean log_2_(I+B/Bud = ∼0)) (Fig. 9, D and F, upper panel). Although reasons for this require investigation, the concept that the independent positive transcriptional effects of RELA and GR, as shown for *BIRC3*, integrate to offset a more generalized, and potentially distinct, glucocorticoid-dependent repression that occurs in the context of the combination treatment is plausible.^23^

## 4 Discussion

Glucocorticoid- and cytokine-dependent upregulation of BIRC3 expression was documented in pulmonary epithelial cells.^8^^,^^9^ Conversely, BIRC2 mRNA was increased by IL-1*β* and TNF*α* in A549 cells; however, protein expression remained unchanged by IL-1*β*, TNF*α*, and/or glucocorticoids in A549, BEAS-2B, or primary human airway epithelial cells.^9^ As a consequence, BIRC2 mRNA upregulation has unclear significance, but could facilitate protein replacement following signal-induced degradation. Notwithstanding possible functions for these 2 BIRC genes, roles for GR and NF-*κ*B in regulating BIRC3 and BIRC2 expression are strongly supported.^9^ Further, glucocorticoid-dependent binding of GR to 1 primary region, plus multiple minor regions, and IL-1*β*- or TNF*α*-activated recruitment of RELA to 2 main regions was demonstrated at the *BIRC3*/*BIRC2* locus. Accordingly, GR and RELA recruitment to R2 and R4, upstream of the *BIRC3* TSS, are consistent with direct regulation by both factors. Likewise, IL-1*β*-/TNF*α*-induced RELA recruitment to R7, at the *BIRC2* TSS, is consistent with NF-*κ*B-dependent regulation of BIRC2. Importantly, these effects were highly concordant between A549 and BEAS-2B cells, and public-domain micro-C and Hi-C data indicated DNA looping between many of these regions and each TSS. Further, given that the R2, R3, and R4 DNA regions increased basal reporter activity by 100–200-fold, with ∼50-fold increases for R5, factors binding these regions, combined with DNA looping, may drive basal *BIRC3* transcription. Conversely, regions R1, R2, R7, and R8 more modestly increased basal reporter activity, which suggests lesser roles in constitutive expression. Indeed, R7, proximal to *BIRC2* TSS, may not be solely responsible for basal BIRC2 expression as interactions involving R2–R5 could also be important. Nevertheless, although non-RELA/non-GR binding regions may also play regulatory roles, RNAP2 presence at the *BIRC2* and, to a lesser extent, *BIRC3* TSSs aligns well with the presence of basal gene transcription and expression.

IL-1*β* (A549) or TNF*α* (BEAS-2B) treatments increased RNAP2 recruitment to each TSS and, in BEAS-2B cells, this correlated with bidirectional run-on transcripts. Thus, NF-*κ*B, or at least RELA, binding at R4 and R7 may promote RNAP2 recruitment to drive *BIRC3* and *BIRC2* transcription, and indeed, the R4 and R7 luciferase reporters both responded to IL-1*β* and TNF*α*. Further, IL-1*β*-induced transcription was attenuated by NF-*κ*B inhibition and NF-*κ*B motif deletion prevented transcriptional drive. Thus sequence-specific enhancers responding to NF-*κ*B are indicated for each region. Despite this, the IKK*β*-selective inhibitors (PS-1145 and ML120B) were poorly effective on IL-1*β*-induced R4 activity. Conversely, TPCA-1, which is weakly selective for IKK*β* relative to IKK*α*,^53^^,^^66^ revealed robust and potent inhibition that is consistent with a possible IKK*α* role. As inhibition of IL-1*β*-induced R7 reporter activity by all 3 IKK inhibitors occurred over an extended concentration range, the data, although consistent with roles for IKK*β*, also suggests additional pharmacological targets that compromise interpretation. Such concerns, as previously raised,^57^ may contribute to disconnects between reporter data and the weak inhibition of IL-1*β*-induced BIRC2 mRNA (which may also be regulated by other factors). Conversely, BIRC3 mRNA was inhibited by PS-1145 and ML120B in a manner consistent with IKK*β*-inhibition, where the greater inhibition by TPCA-1 is readily explained by dual-IKK*β*/IKK*α* inhibition.^58^

Turning to glucocorticoids, these induced BIRC3 mRNA by ∼3-fold,^9^ which, as reported in airways smooth muscle,^26^ is consistent with presence of the upstream R2 GBR. Furthermore, this region, along with R4 and R5, which more weakly recruited GR, drove glucocorticoid-inducible reporter activity that was antagonized by Org34517. With the most glucocorticoid-inducible construct, R5, glucocorticoid-induced transcription required a centrally positioned classical GRE. This contrasts with R2, which had no consensus GRE motif (JASPAR score ≥300) and instead revealed an AR/MR motif. These are similar to GREs, but despite its deletion profoundly reducing overall R2 reporter drive, glucocorticoid inducibility remained. Thus, although R2 recruited GR and reporter activity involved GR, roles for the AR/MR motif in binding of and activation by GR remain unclear. Indeed, that this motif is peripheral to the summit of GR binding peak raises the prospect of a more centrally located, but unconventional, mechanism of GR recruitment and action. Similarly, despite modest GR recruitment to R4 fitting with glucocorticoid-inducible reporter activity that was prevented by Org34517, no GREs were apparent. Rather, this region recruited RELA and deleting any of the 3 RELA motifs prevented glucocorticoid-induced reporter drive. Thus, unconventional GR binding at R4 is plausible. Nevertheless, glucocorticoid-induced DNA looping that involves R2, R4, R5, other promoter elements, and the *BIRC3* TSS may combine to explain overall glucocorticoid-inducible expression.

With glucocorticoid plus IL-1*β*, or TNF*α*, the GR and RELA recruitment patterns were largely similar to the individual treatments. However, at R1 and R2, RELA recruitment by IL-1*β*, or TNF*α*, only occurred in the presence of glucocorticoid. Equally, glucocorticoid-induced R2 activity was enhanced by IL-1*β* without altering glucocorticoid sensitivity and because IL-1*β* alone was without effect, this response represented positive cooperativity. Further, as RELA recruitment to these regions was not associated with RELA motifs, mechanisms for glucocorticoid-permissive RELA binding are unclear. Nevertheless, reduced R2 reporter activity with I*κ*B*α*ΔN, or the IKK*α*/*β* inhibitor, TPCA-1, support NF-*κ*B-dependence. At the IL-1*β*-/TNF*α*-induced R4 RBR, glucocorticoid-dependent GR recruitment also occurred in the presence of IL-1*β*, or TNF*α*, whereas IL-1*β*-induced RELA recruitment was unaffected by glucocorticoid. This explains R4 reporter responsiveness where glucocorticoid- and IL-1*β*-inducibility was additive, without sensitivity changes on combination treatment. However, RELA2 and RELA3 motif deletion prevented glucocorticoid-dependent enhancement of IL-1*β*-induced transcription and this response was antagonized by Org34517. Thus, GR-dependent enhancements at R4 appear to involve unconventional GR-binding at, or around, these NF-*κ*B motifs. This contrasts with R7 where IL-1*β*- or TNF*α*-induced RELA recruitment drove NF-*κ*B-dependent transcription via NF-*κ*B motifs. As this was reduced by glucocorticoid, the transcriptional behavior of this region aligns with IL-1*β*- and TNF*α*-induced BIRC2 mRNA being reduced by glucocorticoid.^9^ Further, the modest GR recruitment to this region could be considered consistent with mechanisms of direct GR-mediated repression.^67^ However, in A549 cells, GR recruitment to R7 was weak, whereas in BEAS-2B cells, GR recruitment in the presence of TNF*α*-plus-dexamethasone was more apparent; however, glucocorticoids did not repress BIRC2 expression.^9^ Thus, GR recruitment to the R4 and R7 RBRs in the presence of an inflammatory stimulus is not supported as a mechanism for transcriptional repression. Rather, R4 clearly shows GR acting at, or near, NF-*κ*B motifs and positively affecting transcription.

Negative glucocorticoid/IL-1*β* interactions occurred at R5. Here glucocorticoid-induced GR- and GRE-dependent transcription were reduced by IL-1*β* in a manner that reflected the reduced GR recruitment, without RELA presence. Although GR loss may represent a relative failure by R5 to compete for nuclear GR, for example, because of mass action consequent on enhanced GR recruitment at other locations (including R8),^21^^,^^22^^,^^39^^,^^68^ an alternative mechanism could involve signaling-dependent effects that then lead to reduced GR recruitment.

Moving to R8, positive cooperativity between glucocorticoid and inflammatory stimuli was shown and this was associated with a modestly increased sensitivity to the glucocorticoid. As RELA was only recruited with glucocorticoid cotreatment and GR recruitment was enhanced by IL-1*β*, or TNF*α*, cotreatment, changes in recruitment may largely explain the observed transcriptional effects. However, as GRE and NF-*κ*B motifs were essential for full reporter activity, it is unclear why factor recruitment, and transcriptional drive, required cotreatment. Could each factor remodel the local chromatin to enhance binding by the other? Alternatively, could signaling mechanisms facilitate factor recruitment? Although answers remain unclear, tonic repression, for example, by p50 homodimers,^69^ which accounts for basal NF-*κ*B DNA binding in A549 cells,^70^ could explain the enhanced basal and glucocorticoid-induced reporter activity by I*κ*B*α*ΔN. Nevertheless, R8 is one of many loci where GR-RELA/NF-*κ*B interaction elicits positive synergy.^22^^,^^64^^,^^71^ However, although micro-C indicated DNA looping between R8 and R7, and other parts of the *BIRC3*/*BIRC*2 locus, this was not confirmed by Hi-C in glucocorticoid-treated A549 cells. The relationship between transcriptional control at R8 and *BIRC3*/*BIRC2* gene expression therefore remains unclear.

In conclusion, positive GR/RELA interaction, presence of both factors with no interaction, and loss of binding and transcriptional activity associated with presence of just one factor on combination treatment are all described at the *BIRC3*/*BIRC2* locus. The data support direct roles for NF-*κ*B and GR in upregulating BIRC3 expression and for NF-*κ*B in upregulating BIRC2. In each case, this presumably helps to maintain antiapoptotic activity, for example, in the context of inflammatory stimuli. Further experiments are needed to explore this. These studies also illustrate how gene transcription may not be due to single transcription factor-binding regions, but may rather entail combinatorial, or integrated, effects involving multiple regions that involve DNA looping. Further, the data provide evidence that GR and RELA interact in a positive and often combinatorial manner. This can involve RELA being recruited to GBRs as well as increased GR recruitment at RBRs. In each case, the recruitment of one factor to the other may not adhere to conventional mechanisms of binding to DNA and this suggestion therefore requires further investigation. More importantly, the overall functional significance of these findings becomes clear with the realization that numerous genes involved in apoptotic control are coregulated by inflammatory cytokines and glucocorticoid. Thus, the recruitment of RELA and/or GR each associate with transcriptional upregulation that in the context of IL-1*β*- or TNF*α*-plus-glucocorticoid, would, as is shown for *BIRC3*/*2*, enable combinatorial effects on the transcription of many genes involved in the control of apoptosis. In these instances, enhancer activity from multiple DNA regions may functionally integrate to control target gene transcription. Finally, combinatorial effects of GR and NF-*κ*B acting directly at gene loci could, in the context of inflammatory stimuli plus glucocorticoid, represent a paradigm to maintain gene expression and, in the case of *BIRC3* and *CFLAR*, promote antiapoptotic function. However, because GO enrichment analyses are necessarily crude and individual genes could be either proapoptotic or antiapoptotic, these data should be viewed as hypothesis generating. Further studies are now essential to explore the downstream consequences of such coregulatory effects on gene expression in relevant functional contexts.

## Conflict of interest

The authors declare no conflicts of interest.
