# Supplementary material for: Differential RELA and GR recruitment to the BIRC3/BIRC2 locus: Molecular insight as to combinatorial regulation by proinflammatory cytokines and glucocorticoid
Source: Mol Pharmacol. 2025 Sep 10;107(10):100073. doi: 10.1016/j.molpha.2025.100073 (PMC12799407; doi:10.1016/j.molpha.2025.100073)
Supplement: Supplementary Tables 1-3 and Supplementary Figures 1-8 [file mmc1.pdf]

Supplemental data:

Differential RELA and GR recruitment to the *BIRC3*/*BIRC2* locus: Molecular insight as to combinatorial regulation by pro-inflammatory cytokines and glucocorticoid

Andrew J. Thorne, Alex Gao, Amandah Necker-Brown, Akanksha Bansal, Keerthana Kalyanaraman, Priyanka Chandramohan, Sarah K. Sasse, Anthony N. Gerber, Mahmoud M. Mostafa and Robert Newton

Department of Physiology & Pharmacology and Lung Health Research Group, Snyder Institute for Chronic Diseases, Cumming School of Medicine, University of Calgary, AB, Canada (A.J.T, A.G., A.N-B., A.B., M.M.M., K.K., P.C., R.N.); Department of Medicine, National Jewish Health, Denver, CO (S.K.S); Department of Medicine and Department of Immunology and Genomic Medicine, National Jewish Health, Denver, CO and Department of Medicine, University of Colorado, Aurora, CO (A.N.G).

Running title: Regulation of BIRC3 and BIRC2 expression by NF-κB and GR

Correspondence: Robert Newton (**rnewton@ucalgary.ca**)

Supplemental Tables

SUPPLEMENTAL TABLE 1

Effect of budesonide and IL-1β on potency and efficacy at the R2, R4, R5 and R8 BIRC3/BIRC2 reporters

Data from the concentration responses analyses in supplemental figure 3 were subjected to four-parameter curve fitting using GraphPad Prism v11 to produce overall pEC_{50} and E_{Max} values, each with an associated standard error (SE).

Reporter	Treatment									
	Budesonide					IL-1β				
	Naïve		+ IL-1β (1ng/ml)		*N*	Naïve		+ Budesonide (300 nM)		*N*
	E_{Max} (Fold [a] ±SE)	pEC_{50} (-log[M] ±SE)	E_{Max} (Fold [a] ±SE)	pEC_{50} (-log[M] ±SE)		E_{Max} (Fold [a] ±SE)	pEC_{50} (-log[g/ml] ±SE)	E_{Max} (Fold [a] ±SE)	pEC_{50} (-log[g/ml] ±SE)	
R2	2.4 ±0.1	8.46 ±0.17	3.3 ±0.1	8.23 ±0.08	5	n.d.	n.d.	n.d.	n.d.	-
R4	n.d.	n.d.	n.d.	n.d.	-	3.6 ±0.1	10.63 ±0.08	4.8 ±0.1	10.45 ±0.07	10
R5[a]	92.2% ±2.80	8.18 ±0.05	9.3 % ±0.5	7.91 ±0.07	5	n.d.	n.d.	n.d.	n.d.	-
R8	2.2 ±0.1	7.89 ±0.10	16.2 ±1.2	8.28 ±0.19	7	n.r.	n.r.	9.3 ±0.2	10.47 ±0.06	6

n.r. = no significant response

n.d. = not determined

[a] R5 reporter data are expressed as % of 1 μM dexamethasone (16.58±1.95-fold).

SUPPLEMENTAL TABLE 2

GO enrichment analysis of DEGs upregulated by IL-1β, budesonide or IL-1β-plus-budesonide.

Gene lists for all DEGs upregulated at any time point by IL-1β, budesonide or IL-1β-plus-budesonide were submitted for enrichment analysis. The top 10, or all, GO terms for biological process or KEGG pathway showing enrichment are shown. Bold indicates the four terms used in downstream analyses. DEG count (Count), fold enrichment (Fold) and Benjamini significance (P_B) are shown.

Treatment	ID	Term	Count	Fold	P_B
IL-1β	**GO:0006915**	**Apoptotic process**	**86**	**2.2**	**1.2E-09**
	**hsa04210**	**Apoptosis**	**32**	**2.8**	**3.0E-06**
	**GO:0043066**	**Negative regulation of apoptotic process**	**61**	**1.9**	**0.00040**
	**GO:0043065**	**positive regulation of apoptotic process**	**44**	**2.1**	**0.00046**
	GO:0006919	Activation of cysteine-type endopeptidase activity involved in apoptotic process	18	3.5	0.00078
	GO:2001240	~negative regulation of extrinsic apoptotic signaling pathway in absence of ligand	11	4.6	0.0053
	GO:0097191	Extrinsic apoptotic signaling pathway	13	3.8	0.0067
	GO:0070059	Intrinsic apoptotic signaling pathway in response to endoplasmic reticulum stress	10	4.4	0.015
	GO:2001238	Positive regulation of extrinsic apoptotic signaling pathway	9	4.6	0.023
	GO:2000427	Positive regulation of apoptotic cell clearance	5	9.6	0.038
Budesonide	**GO:0043065**	**Positive regulation of apoptotic process**	**32**	**2.2**	**0.021**
	**GO:0006915**	**Apoptotic process**	**48**	**1.8**	**0.027**
	**GO:0043066**	**Negative regulation of apoptotic process**	**36**	**1.6**	**0.33**
	GO:0043277	Apoptotic cell clearance	4	4.8	0.83
	GO:0043027	Cysteine-type endopeptidase inhibitor activity involved in apoptotic process	4	4.1	1
	GO:2000271	Positive regulation of fibroblast apoptotic process	3	6.2	1
	GO:0097191	Extrinsic apoptotic signaling pathway	6	2.6	1
	**hsa04210**	**Apoptosis**	**12**	**1.7**	**0.70**
IL-1β + budesonide	**GO:0006915**	**Apoptotic process**	**94**	**2.3**	**2.3E-11**
	**GO:0043065**	**Positive regulation of apoptotic process**	**52**	**2.4**	**2.7E-06**
	**hsa04210**	**Apoptosis**	**28**	**2.5**	**0.00019**
	GO:0006919	Activation of cysteine-type endopeptidase activity involved in apoptotic process	17	3.2	0.0054
	GO:0097191	Extrinsic apoptotic signaling pathway	13	3.6	0.011
	**GO:0043066**	**Negative regulation of apoptotic process**	**57**	**1.7**	**0.012**
	GO:0008630	Intrinsic apoptotic signaling pathway in response to DNA damage	12	3.5	0.024
	GO:0030154	GO:0042981~regulation of apoptotic process	29	1.9	0.054
	GO:0001666	GO:0097192~extrinsic apoptotic signaling pathway in absence of ligand	9	3.7	0.076
	GO:0042311	GO:2001240~negative regulation of extrinsic apoptotic signaling pathway in absence of ligand	9	3.6	0.12

SUPPLEMENTAL TABLE 3

DEGs associated with the biological process terms "*apoptotic process*", "*Positive regulation of apoptotic process*", "*Negative regulation of apoptotic process*", and the KEGG pathway term, "*Apoptosis*".

Term	Treatment	Genes	Count
Apoptotic process (GO:0006915)	IL1B	ADORA2A, BBC3, BIK, BIRC2, BIRC3, CARD16, CASP1, CASP10, CASP4, CASP7, CD14, CD47, CFLAR, CHST11, CSRNP1, CTSC, CTSS, CXCR4, DAPK3, DDIT4, DRAM1, EVA1A, IER3, IFI27, IFI6, IL1A, IL1B, IRF1, JAK2, LCN2, MARCKS, MX1, NFKB1, NFKBIA, NKX3-1, NLRP3, NR4A1, NTN1, NUAK2, PHLDA1, PIM1, PIM3, PLK3, PLSCR1, PMAIP1, PML, PPARD, PPP1R15A, PRUNE2, PSEN1, PTPRH, RASSF5, RELT, RFFL, RHOB, RIPK2, RNF144B, S100A8, S100A9, SAV1, SHB, SRGN, STK17A, TCIM, TEX11, TGFBR1, TNFAIP1, TNFAIP3, TNFAIP8, TNFRSF10A, TNFRSF10B, TNFRSF11B, TNFRSF12A, TNFRSF9, TNFSF10, TNFSF14, TRAF1, TRAF3, UBE2Z, UNC5B, UNC5D, XAF1, YBX3, ZC3H12A, ZFP36L1, ZNF385A	86
	Bud	ADAMTSL4, ADORA2A, AIPL1, ALOX15B, BEX2, BFAR, BIRC3, CASP1, CIDEC, CITED1, DDIT4, EMP1, EMP2, ERRFI1, FAIM, FOXO1, GADD45A, GADD45B, GDF6, GRAMD4, HRK, KANK2, KCNIP3, KCNJ11, KCNJ8, NFKBIA, NOTCH2, NOTCH2NLB, NOTCH2NLC, PIM3, PPP1R13L, PRKCD, PTK2B, RHOB, SEPTIN4, SFRP5, SGK1, SRGN, STK17B, TGFBR1, THBS1, TNFAIP3, TNFSF14, TRAF1, UNC5A, UNC5B, ZBTB16, ZC3H12A	48
	IL1B + Bud	ADAMTSL4, ADORA2A, AIPL1, ALOX15B, BCL2L15, BIK, BIRC2, BIRC3, BOK, CARD16, CASP1, CASP4, CASP7, CFLAR, CIDEC, CSRNP1, CTSS, CXCR4, DDIT4, DRAM1, EMP1, EMP2, ERRFI1, FAIM, FOXO1, GADD45A, GADD45B, GADD45G, GRAMD4, HRK, IER3, IFI27, IL1A, IL1B, IRF1, JAK2, KANK2, KCNJ11, LCN2, MX1, NFKB1, NFKBIA, NKX3-1, NLRP3, NOTCH2NLB, NOTCH2NLC, NR4A1, NTN1, NUAK2, PHLDA1, PIM3, PLK3, PLSCR1, PMAIP1, PML, PPP1R13L, PPP1R15A, PRKCD, PRUNE2, PSEN1, PTK2B, RASSF5, RELT, RFFL, RHOB, RNF152, S100A8, S100A9, SAV1, SEPTIN4, SFRP5, SGK1, SGMS1, SHB, SRGN, STK17B, TEX11, TGFBR1, TLR2, TNFAIP3, TNFAIP8, TNFRSF10B, TNFRSF9, TNFSF14, TNIP2, TP73, TRAF1, UNC5A, UNC5B, VEGFA, XAF1, ZBTB16, ZC3H12A, ZFP36L1	94
Positive regulation of apoptotic process (GO:0043065)	IL1B	ATF4, ATG7, B4GALT1, BBC3, BCL2A1, BID, BMP2, BMP7, DAPK3, DNAJA1, DUSP6, FOSL1, FZD9, GAL, IDO1, IFIT2, IL6, IRF8, JUN, KCNMA1, MCL1, NR4A1, OLFM1, PHLDA1, PMAIP1, PSEN1, PTGS2, RHOB, RIPK2, RPS6KA2, SAV1, STK17A, TGFBR1, TGM2, TLR3, TNF, TNFAIP8, TNFRSF10A, TNFRSF10B, TNFRSF12A, TNFSF10, UBD, UBE2Z, WT1	44
	Bud	ACE, ADAMTSL4, ANKRD1, B4GALT1, BCL6, CLIP3, CNR1, DCUN1D3, DUSP6, FOXO1, FOXO3, FRZB, GADD45A, GADD45B, HPGD, HRK, KCNMA1, KNG1, MCL1, MST1, NEURL1, NOTCH2, NOTCH2NLB, NOTCH2NLC, RHOB, RPS6KA2, SEPTIN4, STK17B, TGFBR1, TGM2, TXNIP, ZBTB16	32
	IL1B + Bud	ADAMTSL4, B4GALT1, BCL2A1, BCL6, BID, BMP2, BOK, CNR1, CTNNB1, DCUN1D3, DUSP6, FOSL1, FOXO1, FOXO3, FZD9, GADD45A, GADD45B, GADD45G, GAL, HPGD, HRK, IDO1, IFIT2, IL6, IRF8, JUN, KNG1, MCL1, NEURL1, NOTCH2NLB, NOTCH2NLC, NR4A1, PHLDA1, PMAIP1, PSEN1, PTGS2, RHOB, RPS6KA2, SAV1, SEPTIN4, STK17B, TGFBR1, TGM2, TLR3, TNF, TNFAIP8, TNFRSF10B, TP73, TXNIP, UBD, WT1, ZBTB16	52
Negative regulation of apoptotic process (GO:0043066)	IL1B	AKR1B1, ANGPTL4, ASNS, BCL2A1, BCL3, BIRC2, BIRC3, CD38, CD44, CFLAR, CHST11, CX3CL1, DNAJA1, DNAJC3, EGFR, EGR3, EIF2AK2, FSTL1, GATA6, HCK, HMGA2, HPN, IER3, IFI6, IFIT3, IL6, IL7, MAP4K4, MCL1, MSX1, MSX2, NFKB1, NUAK2, PIM1, PIM3, PLAUR, PLK2, PLK3, PPIF, PSEN1, RELA, RNF144B, SERPINB9, SMAD3, SOCS2, SOCS3, SOD2, SOX9, SPHK1, STK40, TCIM, TEX11, TFRC, TGFBR1, TGM2, TNF, TNFAIP8, TNFSF18, UBE2Z, WNT5A, WT1	61
	Bud	AIPL1, ANGPTL4, BFAR, BIRC3, CD38, CDKN1A, CITED2, CLDN7, CRYAB, DPEP1, EGFR, FMN2, FOXO1, GOLPH3, HCK, HSPB1, KRT18, LIMS2, MCL1, NIBAN2, NOL3, NOTCH2, NOTCH2NLB, NOTCH2NLC, NR1H4, NUPR1, OSR1, PAX7, PIM3, PTK2B, SHC1, TBX3, TENT5B, TGFBR1, TGM2, THBS1	36
	IL1B + Bud	AIPL1, ANGPTL4, BCL2A1, BCL3, BIRC2, BIRC3, CD38, CFLAR, CITED2, CLDN7, CRYAB, CTNNB1, CX3CL1, EGFR, EGR3, FOXO1, GATA6, HCK, HMGA2, HSPB1, IER3, IFIT3, IHH, IL6, IL7, KRT18, MCL1, MSX1, MYC, NFKB1, NIBAN2, NOTCH2NLB, NOTCH2NLC, NUAK2, PAX7, PIM3, PLK3, PSEN1, PTK2B, SCX, SERPINB9, SOCS3, SOD2, SOX8, SOX9, SPHK1, TBX3, TENT5B, TEX11, TGFBR1, TGM2, TNF, TNFAIP8, TNFSF18, VEGFA, WNT5A, WT1	57
Apoptosis (hsa04210)	IL1B	AIPL1, ANGPTL4, BCL2A1, BCL3, BIRC2, BIRC3, CD38, CFLAR, CITED2, CLDN7, CRYAB, CTNNB1, CX3CL1, EGFR, EGR3, FOXO1, GATA6, HCK, HMGA2, HSPB1, IER3, IFIT3, IHH, IL6, IL7, KRT18, MCL1, MSX1, MYC, NFKB1, NIBAN2, NOTCH2NLB, NOTCH2NLC, NUAK2, PAX7, PIM3, PLK3, PSEN1, PTK2B, SCX, SERPINB9, SOCS3, SOD2, SOX8, SOX9, SPHK1, TBX3, TENT5B, TEX11, TGFBR1, TGM2, TNF, TNFAIP8, TNFSF18, VEGFA, WNT5A, WT1	32
	Bud	BIRC3, CAPN2, FOS, GADD45A, GADD45B, HRK, ITPR3, MCL1, NFKBIA, SEPTIN4, TRAF1, TUBA3E	12
	IL1B + Bud	BCL2A1, BID, BIRC2, BIRC3, CASP7, CFLAR, CTSS, EIF2AK3, FOS, GADD45A, GADD45B, GADD45G, HRK, ITPR2, ITPR3, JUN, MAP2K1, MAP2K2, MCL1, NFKB1, NFKBIA, PMAIP1, SEPTIN4, TNF, TNFRSF10B, TRAF1, TRAF2, TUBA3E	28

Supplemental Figures

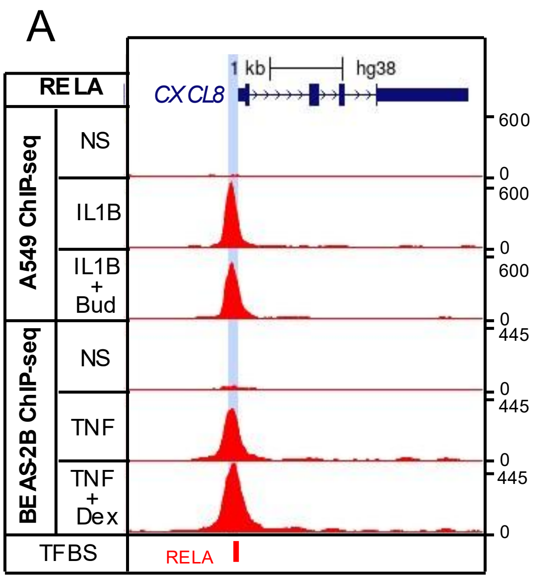

Supplemental Figure 1. ChIP-seq traces showing RELA and GR recruitment to the *CXCL8* and *FKBP5* loci. Genome browser snapshots of A549 and BEAS-2B cells treated with IL-1β (1 ng/ml) and/or budesonide (Bud; 300 nM), or TNFα (10 ng/ml) and/or dexamethasone (Dex; 100 nM), respectively. Arrow heads within the intronic regions indicate direction of transcription. (A) RELA ChIP-seq traces (red) at the *CXCL8* locus. (B) GR ChIP-seq traces (black) at the *FKBP5* locus. Highlighted regions (light blue) correspond to GR and/or RELA binding positions as detected by qPCR in the current study. Transcription factor binding sites (TFBS) for RELA motifs (red boxes) and GREs (black boxes), as predicted from the JASPAR CORE database (score ≥ 300), are indicated.

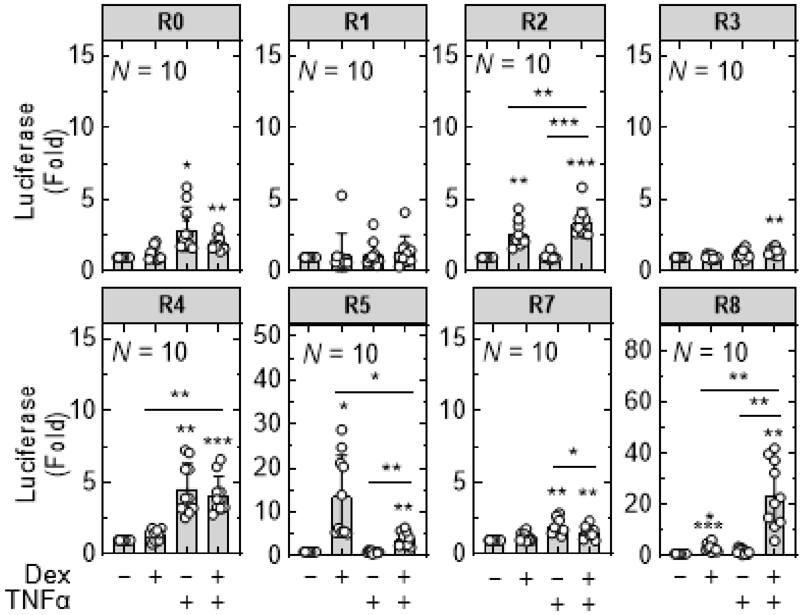

Supplemental Figure 2. Transcriptional activity of GR- and RELA-binding regions at the *BIRC3*/*BIRC2* locus following TNFα and dexamethasone treatments. The regions R0 – R8, as highlighted in Fig 2A, were cloned into a TATA-containing luciferase vector prior to stable transfected into A549 cells. R0 – R8 reporter constructs were either not stimulated or stimulated with dexamethasone (Dex; 1 μM), TNFα (10 ng/ml), or both. Cells were harvested after 6 h and luciferase activity assessed. Data from *N* independent experiments were plotted as fold of no stimulation ±SD. Data, expressed as mean ± S.D., and are shown as bar graphs overlaid with scatter plots. Significance was tested by one-way ANOVA with a Tukey's post-hoc test. * $P \leq 0.05$, ** $P \leq 0.01$, *** $P \leq 0.001$ indicates significance to not stimulated or as indicated.

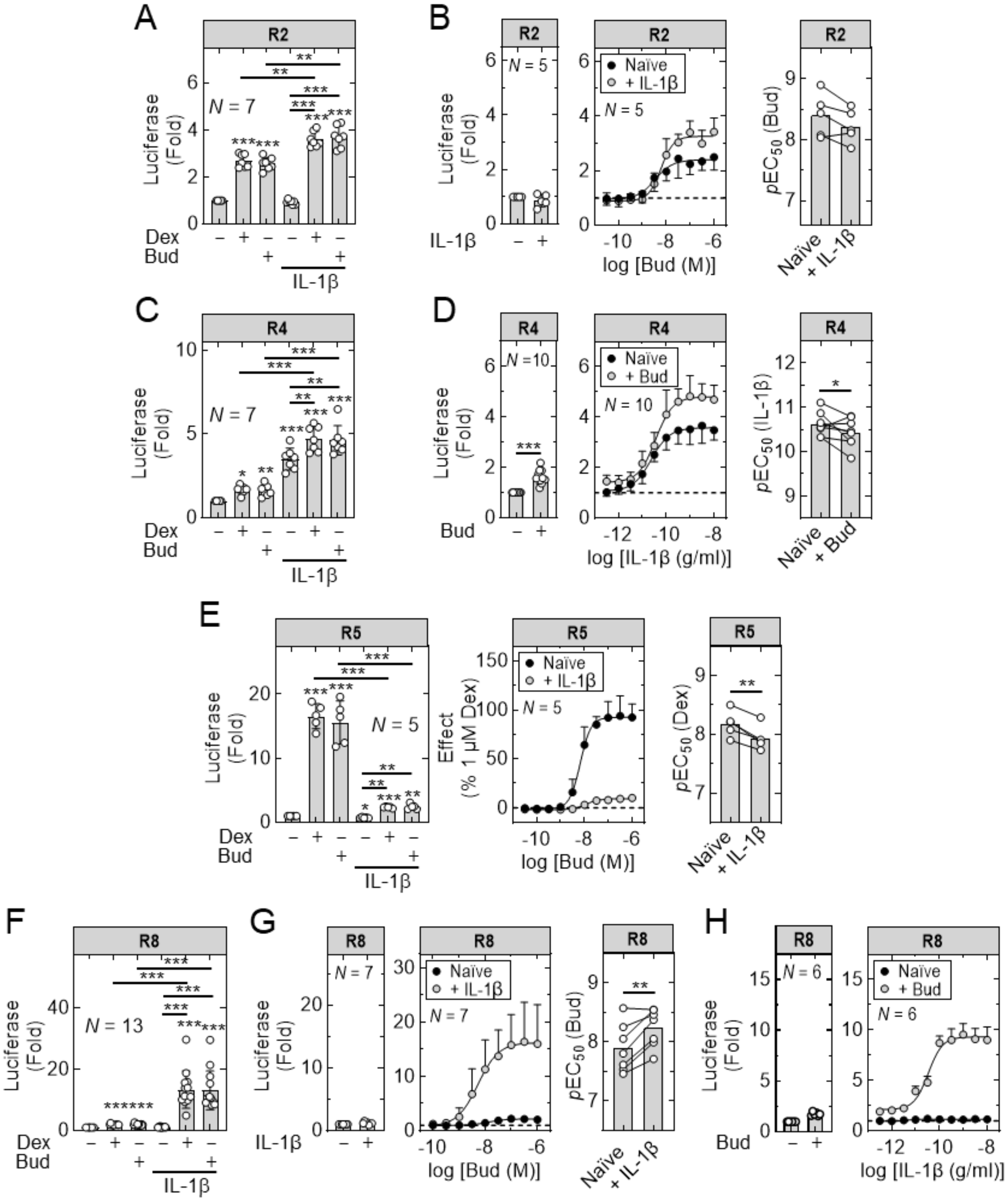

Supplemental Figure 3. Transcriptional activity of GR- and RELA-binding regions at the *BIRC3*/*BIRC2* locus following treatment with IL-1β and/or budesonide plus comparison with dexamethasone. The regions R0 – R8, as highlighted in Fig 2A, were cloned into a TATA-containing luciferase vector prior to stable transfection into A549 cells. (A) The R2 reporter was either not stimulated or treated with dexamethasone (Dex, 1 μM) or budesonide (Bud, 300 nM) each in the absence or presence of IL-1β (1 ng/ml). Data were expressed as fold of untreated and are plotted as mean ± SD overlain with scatted plots (B) The R2 reporter was either not stimulated or treated with the indicated concentrations of budesonide each in the absence or presence of IL-1β (1 ng/ml). Data were expressed as fold of untreated and are plotted along with their fitted curves. Using paired data from each individual experiment, curve fitting was performed for each treatment (naïve, +IL-1β) and individual pEC_{50} values plotted (right panel). (C) The R4 reporter was treated as in A. (D) The R4 reporter was either not stimulated or treated with the indicated concentrations of IL-1β each in the absence or presence of budesonide (300 nM). Data were expressed as fold of untreated and are plotted along with their fitted curves. (E) The R5 reporter was

treated as in A and B, but as single combined experiments. Data for untreated, IL-1β (1 ng/ml), dexamethasone (1 μM), budesonide (300 nM) and the indicated combinations were plotted as fold of untreated (left panel). Data for each budesonide concentration series was then expressed as % effect of dexamethasone at 1 μM where no stimulation = 0 and 1 μM dexamethasone = 100 (middle panel) and curves fitted. pEC_{50} values were obtained as in B (right panel). (F) The R8 reporter was treated and graphs plotted as in A. (G) The R8 reporter was treated and graphs plotted as in B. (H) The R8 reporter was treated and graphs plotted as in D.

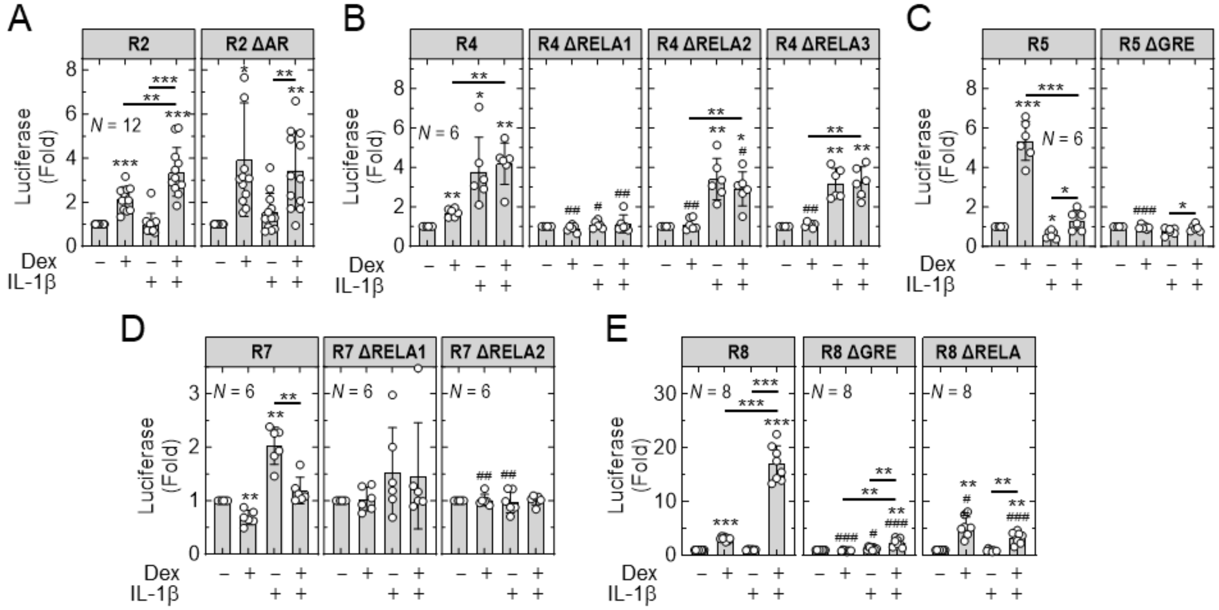

Supplemental Figure 4. Effect of GRE or RELA motif deletion on R2, R4, R5, R7 and R8 transcriptional activity induced by dexamethasone or/and IL-1β showing fold activity of each reporter construct. Each GRE or RELA motif within the R2, R4, R5, R7 and R8 reporters, as depicted in figure 3, was deleted by site-directed mutagenesis to give the respective ΔGRE or ΔRELA constructs. Wild-type and mutated constructs were transfected in parallel into A549 cells prior to G418 selection. Stably transfected cells for: (A) R2 & R2ΔAR; (B) R4, R4ΔRELA1, R4ΔRELA2 & R4ΔRELA3; (C) R5 & R5ΔGRE; (D) R7, R7ΔRELA1 & R7ΔRELA2; and, (E) R8, R8ΔGR & R8ΔRELA were either not stimulated or stimulated with dexamethasone (Dex; 1 µM), IL-1β (1 ng/ml), or both. Experiments were performed in parallel on all constructs for each reporter (R2, R4, R5, R7 & R8) and after 6 h, cells were harvested for luciferase activity determination. Data from the *N* experiments shown in Fig. 5 of the main manuscript were expressed as fold of untreated for each individual construct and were plotted as mean ±SD overlaid with scatter plots. Significance was tested by one-way ANOVA with a Tukey's post-hoc test. For each reporter construct (wild type, ΔGRE or ΔRELA) * $P \leq 0.05$, ** $P \leq 0.01$, *** $P \leq 0.001$ indicates significance to its own not stimulated or as otherwise indicated. # $P \leq 0.05$, ## $P \leq 0.01$, ### $P \leq 0.001$ indicates significance between the fold for each deletion and the wild-type construct for each treatment.

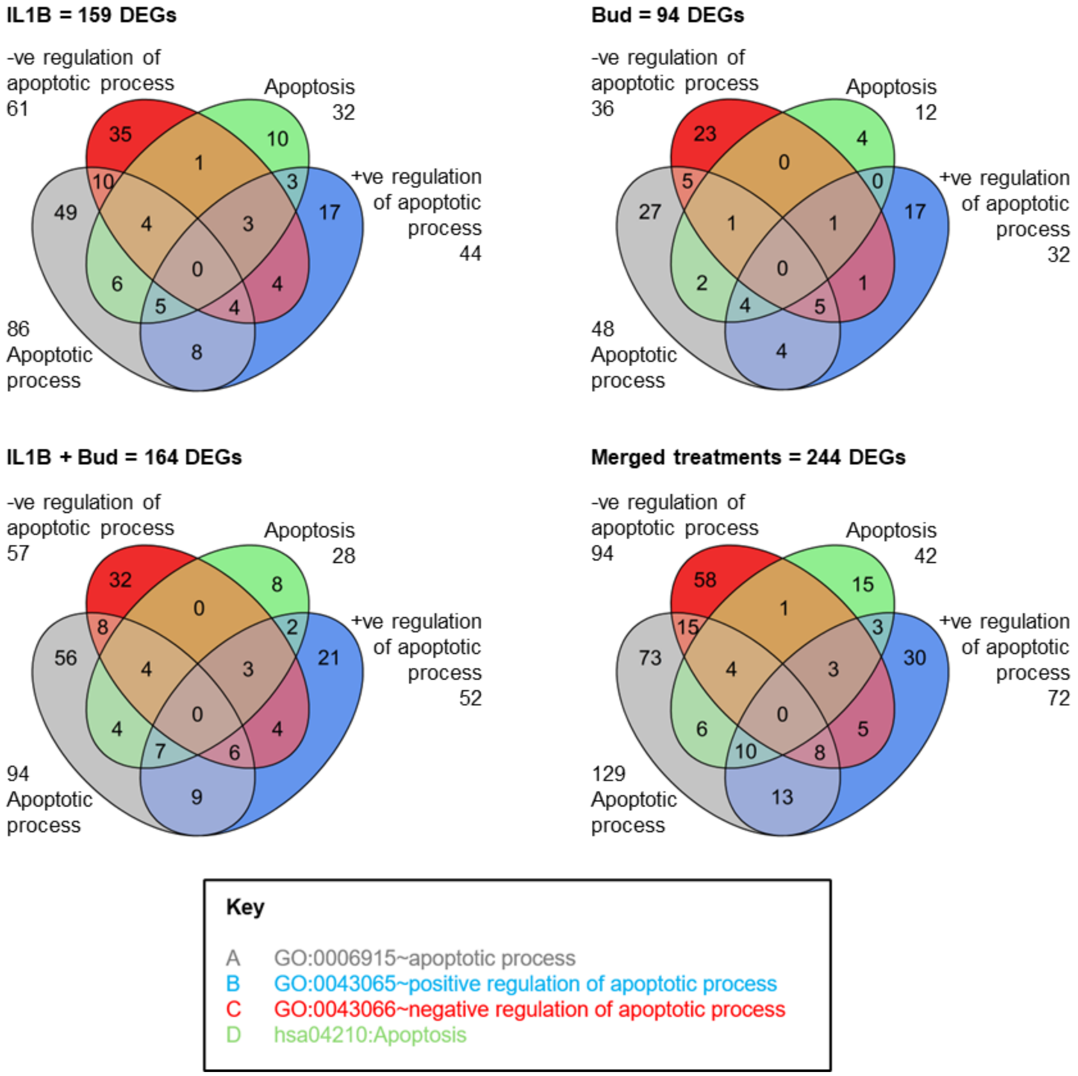

Supplemental Figure 5. DEGs associated with the three biological process terms "*Apoptotic process*", "*Positive regulation of apoptotic process*", "*Negative regulation of apoptotic process*", and the KEGG pathway term, "*Apoptosis*" were plotted as Venn diagrams each of the treatments IL-1β, budesonide (Bud) and IL-1β-plus-budesonide. Total DEG number the numbers in each region/overlap are indicated.

A

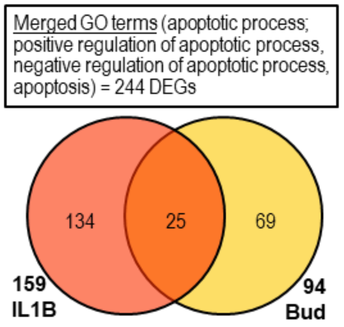

χ^2 ****	IL-1β Not induced	IL-1β Induced	Totals
Bud Not induced	25,412 (O)<br>*25387.6 (E)*	134 (O)<br>*158.4 (E)*	25,546
Bud Induced	69 (O)<br>*93.4 (E)*	25 (O)<br>*0.6 (E)*	94
Totals	25,481	159	25640

(O) = Observed values
(E) = expected values (in *italics*)

B

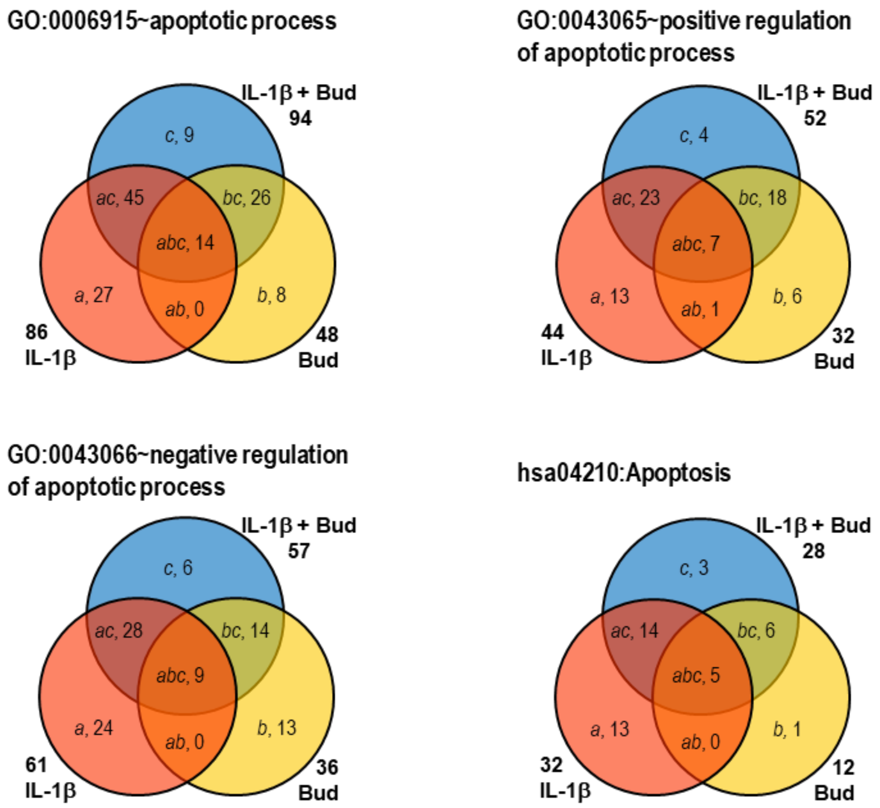

Supplemental Figure 6. Overlap between the DEGs upregulated by IL-1β and budesonide that were associated with the biological process terms "*apoptotic process*", "*Positive regulation of apoptotic process*", "*Negative regulation of apoptotic process*", and the KEGG pathway term, "*Apoptosis*". (A) Venn diagram depicting overlap between the 159 DEGs upregulated by IL-1β and the 94 DEGs upregulated by budesonide is shown. A contingency table to show the observed and expected numbers of DEGs within each category is shown where 25640 is the number of unique NCBI gene IDs included in this analysis. Following Chi^2 testing **** = $P \leq 0.0001$. (B) Venn diagrams show total DEG numbers and overlap for DEGs induced by IL-1β, budesonide for each of the four GO terms selected for downstream analysis.

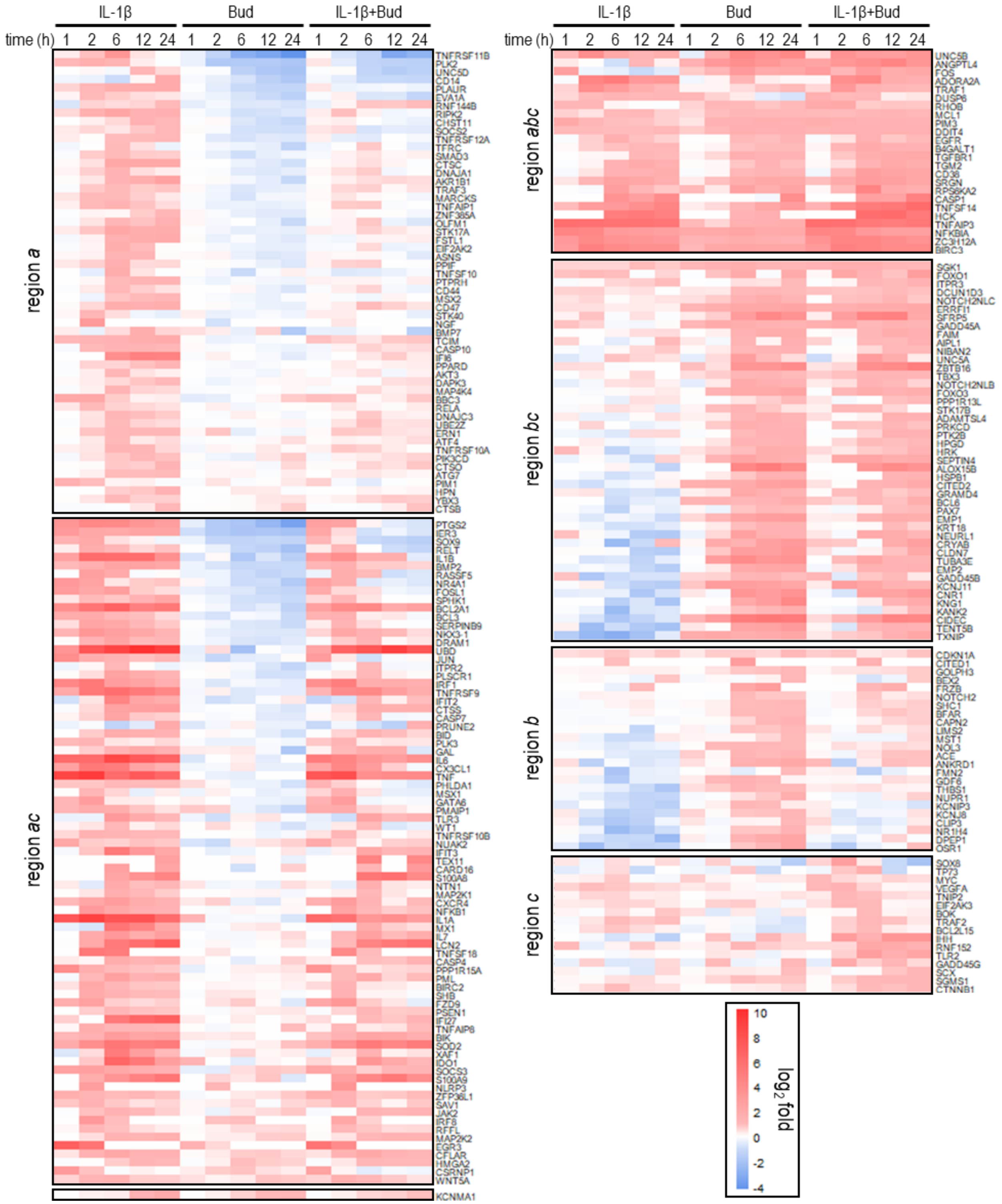

Supplemental Figure 7. Enlarged version of the heap map shown in figure 9B to show gene symbols for each DEG.

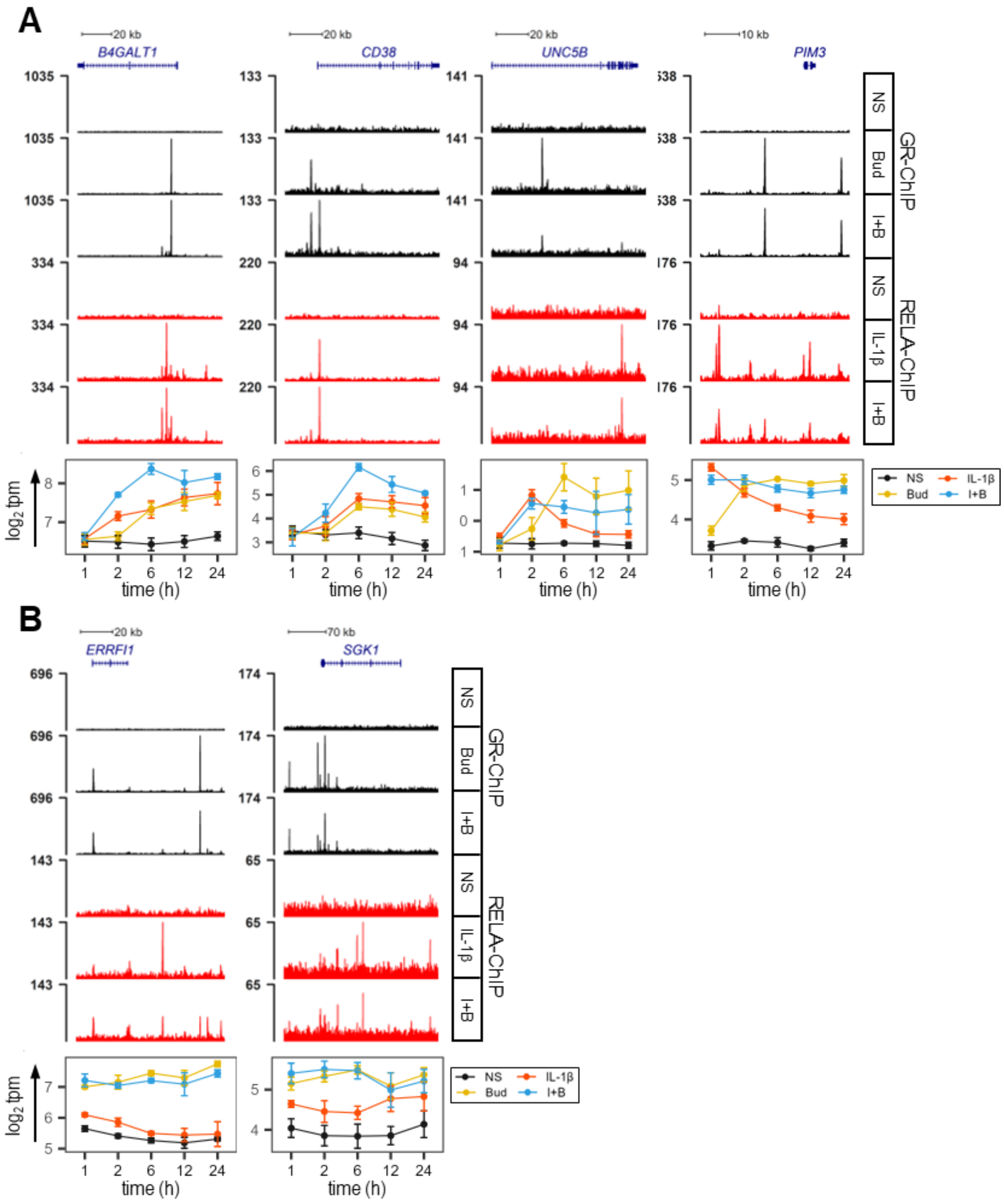

Supplemental Figure 8. GR and RELA ChIP-seq data and mRNA-seq data for: (A) genes (*BAGALT1*, *CD38*, *ERRFI1*, *PIM3*) in region *abc*; and (B) genes (*SGK1*, *UNC5B*) in region *bc* of the Venn diagram in Fig. 9A. ChIP- and mRNA-seq data are as described in figure 9.
